# Supplementary material for: The Vital Role of the CAMTA Gene Family in Phoebe bournei in Response to Drought, Heat, and Light Stress
Source: Int J Mol Sci. 2024 Sep 10;25(18):9767. doi: 10.3390/ijms25189767 (PMC11432206; doi:10.3390/ijms25189767)
Supplement: Supplementary file 1 [file ijms-25-09767-s001.zip › Sequences.pdf]

>PbCAMTA1
MSRYDINKLFQEAQSRWLKPAEVFFVLQNYDKHHLNLKPPETHKVVRYGVSWRKKKDGKT
VGEAHERLKVGDVDALNCYYAHGEQNPNFQRRCYWILDPTCDHIVLVHYREITDGKTYNG
STSNLLPDSFSIFSQNSGYCNVHPGSSSGISELHESDQSSFTPGSVEEASSTFVGRNNNM
DHVDVDRPGEFGSSSYPEVNQALRRLTVQLSLDDDDGLMYLEKLPPDYSQIENPQDSELL
VYGSEESKHDALIGLPLRERDQWSVGNNRVQANSKKLLLQNSGDTEKQHGQPFDPGCKTD
GKEPNSWKEMLEMSSISTGINSQNKSLDILPLNGVSHSPRNEIFKYEAGYHSMDATSLET
GSLLLSDRGNVSTDLAMQPENSIYQWQDFKGMNGANRSNCHRNSESDFSLQLSATRQFLL
GSDDAIISPSSNELTQKDEKSVHSILSSATPTLGTDSCMGMLGRTDSIAWMETGNIPIVK
HTPDCYEVWFNQKNRIATPVEADSSLTTPQEQHFSIREISPGWAYSTESTKLIITGDFLC
DHLEHVWGCMFGDIEVPAEVIQAGVLRCQAPRHKAGKVTFCITSDNKEPCSEVREFEYRM
KLKSSTLDDDLPEKDAYKSTEELLLLAKFAKKLLVGRDSSSVARIDYVESGVGNSRKSRI
ADDPWDQIIESLLIGSENPSTIMDSILQELLKDKLQQWLSSRCRGERSIIHMIAGLGYEW
ALNSILNVGVGINFRDLNGWTALHWAARFGREKMVAALLAAGASAGAVTDPTSQDPVGKS
PASIAAACGHKGLAGYLSEVALTSHLSSLTMKESKISEGSAAVEAERTVESISDKSIQIP
ADAIEDQLSLKDSLAAEKQQRAAASCDEHGMTADDIHGLSAASKFLGLRDRKLNTAALSI
QKKYRGWKGRKEFLALRQNVVKIQAHVRGHQVRRKYKFLWTVGVLEKVVLRWRRKGVGLR
GFRAEPLPIDQSEDDDILKVFRKQKVDVAVDEAVSRVLSIVDSPKARQQYRRILEGYRQA
KAQISSNNGKALSSQGNDRESDVDDMRYFA
>PbCAMTA2
MTTQSGFDISKLFQEAHSRWLKPAEVYYILQNYETCPLTLKAPQNPPSGSLFLFNRRVLR
FFRKDGYSWRKKKDGRTVGEAHERLKVGNVDALNCYYAHGEQNPNFQRRCYWMLDPACDN
IVLVHYREITEGRPNNGSTSHSSPESFQISARVLNIVVLNILAPLLESLKSRGFGFPSYP
EVNQVLQRLTKELSLGNDDSSMYFEKLPPDYSQSESPHDSGFLACESELSKPDERLCLPL
GQKCGEDAQWIIGNDGVQDDAENMLQNSDDTVDQKESLYWKEMLELSSSSTGTGANSQNQ
TSDRLALNGVPNPSTKGIEYEAAHYSKDATSLEHASLSIPERVNSCMDSAAQPEELICQW
QDLEGNDGTNGMNCHQTSKNNFNLDLSAAEQFLLGSDDTIISQASGKFLQDSETSVHSTY
SSGTSTLDTNSGTRMLKKTNSVDWMETRNISIDKYSPDYYEAWFDQESHLGTTTLEADSG
LTIAQKQRFSIREISPEWAYATEGTKLIIVGDFLCDPLECAWGCMVGDIEVPIEVIQAGV
ILCQAPQHTPGKVTLCITSGNRESCSEVREFEYRIKPMSSALESNLPEKDANKSAELLLL
ARFAQILLLGHDSSSVQIESNVEWESNALRKPKMDDDTWGQIMEALLVGSESTSNIMDCI
LQELLKDKLQQWLSSKCHEGEVRASSLSKEEQGIIHMIAGLGYEWALNSILDAGIGINFR
DVKGWTALHWAARFGREKMVAELLAAGAFAGAVTDPTPQDPVGKNPAFIAAACGHKGLAG
YLSEVALTSHLSSLTLKESEISKGSAAVEAERTVEIVSDKSMQIPNSAVEDQLSLKDSLA
AVRNAAQAAARIQAAFRAHSFRQRHLSADASCDEYGLTADDIHGLSAASKLHGLRDHKLN
TAALSIQKKYRGWKRRKEFLTLRQNVVKIQAHVRGHQVRKKYREFLWTVGVLEKAVLRWR
RKGVGLRGFRAEAGPIDESEDDDVLKVFRKQKVDAAVDEAMSRVLSVVESPSARQQYRRL
LESYRRAKAQLSSNIGEMSSSQVNDSMENFTDDEMYYFA
>PbCAMTA3
MQSGGGFDINELRREAQKRWLKPTELLFILQNFDDDQLIHEPPQNPTSGSLFLLNRKVLR
HYRKDGHSWRKKKNGRTVGEAHEHLKVGNTDTLNCYYAHGDQNPNFQRRIYWMLDPACGD

IALVHYREVTEGRYIVGPNLPTDSNPTFSQSTRFGDSQNPDSSDPYQNSVSSHCMDKVSS
KLAVGNNERSFFGVDRLGDVGSSSEPESTLALPRFTEQLSLDGEHSYLAEKLPCCSQNEN
FQEPAFFGSTRAVYGHDNLIDHPLQLQFEEHGLLLDIDIVDTASQQHSQPADQEKISDQV
RQEELPDSAFMFIGGSQAKDMTSHDGLLESLERSKVSYAQDEQPEHLLFHCLDHRLNDAD
YMINTHQTTLDANSGVGMLTKTNSTDWMDTRHVPFDNHKYCSEYSMFDQDSLVGNPLGPD
SSLTVSQMQRFSIREISPEWAYTTGNTKVIITGDFLCDSSDGTWACMFGETEVHAEVIQT
GVLRCRAPPHVAGKVNFCVTAGNRESCSEVREFEYCALPESPGFDNKSPQADTTSKSSAE
LHLLVRFAEMLFFGYDNVSVQKEDSSKSESNPSRNSKLDSNSWRQIIEALSVGRDIPSCM
EIMDWLLQELLKDKLQHWLSSKYQEDEGTICPLSKKEQGVIHMVAGLGYEWALNSVLNSG
VGINFRDGNGWTALHWAARFGREQMVAALVAAGASAVAVTDATSQDPVGKNPASIAASNG
HRGLAGYLSEVALTSHLSLLTLEETERTENIPVGEVEDELSLKDSLAAARNAAQAAARIQ
AAFRARSFRKKRLRELKSYDENGWTLGDIHGLSAAMKFQWAFHKLHNVKLNIAALSIQKK
YRGWKRRREFLTFRKNVVKIQAHVRGHQVRKRYKELLWTVSILEKIILRWRRKGDGLRGF
RGDLESIDEAEDDDVPKVFREQKVHAVIEEAVSRVLSMVECPDARHQYRRILERYRQAKA
EMVHTTSETASSSQANEQIMENDGGDIGRGRPTRPRQITFPVGVRKDDDIDQSELSTETK
ALISSGPVGASAKDEKGSEGAADIGMEEIVKELRVIRKQNTVTHWLLSVLIVTTAVWQMS
EVSLLLYLKDRVKHPFKAIGGLITGSLGRSNESDAAKSNSSSKQHQIESIPLPHQIKMSE
LLSMEMSSLGLNGQSQD
>PbCAMTA4
MESGIQRLAGWDIHGFHTLEDLDIVKTKEEALTRWLRPNEIYAILCNCVYFRINVKPVEL
PQSGTIILYDRKMLRNFRKDGHNWKKKKDGKTVKEAHEHLKVGNQDRIHVYYAHGQDNPN
FVRRIYWLLEKKLEHLVLVHYREVSEDNATSYLAPPNECKEALSLSNRMHHGSPVTPVNS
HSGSIHSETSGSRVVSEEINSGIDHAFHAGSGTSLVGESTKFGSQLDESLIVMDHEQRLR
EINTLDWADLLEANRNPNSAEPGNGEISYFDLQRPYGLSDSRSLIIFFHITIHSLGNPSI
DINGSGYLSAIQPNNGYPVGDQNVQPSLFETIDTNLLKKDAGMATVGAVETSEMFDKNPL
LSQDSLARWMSIIANDSPGSVDEQPMECSISTVNGSAATMVKDQSALQEAFSITEVSPTW
AFSTEETKVIVIGYFHEAHAYLAESNLFCVFGDVYVPAERVQVGVFRCMALPCRAGLVDF
YLSLDGHRPISQVLSFEYRPVSTNQMSNEISVLEDEKSKWRDLQLQIRLAHLLFSTTNNI
AALSSNMPPDVVKEAKKFTVLTPSYEKEWTKFVDLIMNKEMSFAEAHQNFFELTLKNKLQ
EWLLERVAEGCKTTPRDCKGQGVIHLCAMLGYTWAVYPFKLSGLSLDFRDSFGWTALHWA
ACYGREQMVAFLLSAGANPSMVSHSTAKCPGGYTAADIASMKGYDGLAAYLAEKGLTAHF
NAMCTAGNVSGSLRTTATTMEQPENLNEEEQLLRDSLAAYRSAADAAARIQTALREHALK
VRTKVVQLENTETEALNIISAMKIQHAYRNYHSRKRLAAAVRIQHRFRTWKIRKDFLNMR
YQAIRIQAAFRGHLVRRQYHKILWSVGVLEKAILRWRQKRKGLRGLPAEPAIASGAEEVQ
GDDVEEDFYRLSRKQAEERVERSVVRVQAMFRSHQAQKEYRRMKIAFDQAQVEYEGLLGS
PMQE
>PbCAMTA5
MAEGDGRYTREEGENASINSITSENRVTPIWRYSVAVSYSFAYVALEDASWLSSHTCEFA
FGSIHSETSGSRVVSEEINSGIDHAFHAGSGTSLVGESTEFGSQLDESLIVTDHEQRLRE
INTLDWADLLEANRNPNSAEPGNGEISYFDLQRPYGLSDSRSLDHFLPYHNSSSLGNPSI
DINGSGYLSAIQPNNGYPVGDQNVQPSLFETIDTNLLKKDAGMATVGAVETSEMFDKNPL

LSQDSFARWMSIIANDSPGPVPMECSISTVNGSGATMVKDQSALQEAFSITEVSPTWAFS
TEETKVIVIGYFHEAHAYLAESNLFCVFGDVYVPAERVQVGVFRCMALPCRAGLVDFYLS
LDGHRPISQVLSFEYRPVSTNEMSNEISVLEDEKSKWRDLQLQIRLAHLLFSTTNNIAAL
SSNMPPDVVKEAKKFTVLTPSYEKEWMKFVDLIMNKEMSFAEAHQNFFELTLKNKLQEWL
LERVAEGCKTTPRDCKGQGVIHLCAMLGYTWAVYPFKLSGLSLDFRDSFGWTALHWAACY
GREQMVAFLLSAGANPSMVSHSTAKCPGGYTAADIASMKEKGLTAHFNAMCTAGNVSGSL
RTTATTMEQPENLNEEEQLLRDSLAAYRSAADAAARIQTALREHALKVRTKVVQLENTET
EALNIISAMKIQHAYRNYHSRKRLAAAVRIQHRFELGRSAKIFSICATKLLEFKLHSEDT
>PbCAMTA6
MAEGRRYALGTQLDIEQILLEAQSRWLRPPEICEILRNYHKFRIAPEPPNKPPSGSLFLF
DRKVLRYFRKDGHNWRKKKDGKTVKEAHERLKAGSVDVLHCYYAHGEENENFQRRSYWLL
EEDLMHIVLVHYREVKGNKVNYSRTRDSEETYQVPSRTMDTSSLNSVHTSELDDSESDNH
QASSRYCSFLDEQSEDRPVTNDLMDAGLLNSHLPVSYPSDYHGKQSNVPGFNFMTLGQDN
LTRVDGSEFGLTFQDPGKQNGVASWVEALEHTGTQSHTASFRLPVSSTKPTSIMGIPKPE
NVITGHFFTEACGIKQQIVASPLEQAQWQIVAEDDSSNTSTWPMDQNLDTDLAAELSVSL
EERKGHFVDVVDDPASPSVDSDQEHGSFMPNKLQLNLSNADVGGLHAENGQVREENFSYS
SSSRHHLIDCSKKAEDDLKKLDSFSRWMSKELEVDGAHVQSSSGIDWNTVENGNVAESGM
SAQGHLDAYLLSPSRSQDLLFSILDFSPNWVYAGMETKVLVTGMFLRDKNDAQICKWSCM
FGGVEVPAEVLADGVLRCRAPVHVAGRVPFYVTCSNRVACSEVREFEYRIGHTRSVDTSD
TNNGNINEMFLRIRLGKLLSLDSIGRPKPLSSNVGAKSNVNSKISSLLKDNDDEWLQIEK
LTTDEDFCPDKVEELLLQKILKEKLHTWLLHKVAEDGKGPNVLDKEGQGVLHLAAALGYD
WAIAPTIAAGVNINFRDVDGWTALHWAAFCGRERTVVALVSLGGAPGALTHPTPKFPPGR
TPADLASSNGHKGIAGYLAESSLTSHLSSLTLETSSDGNASEVPDSKAVQTVAERSATQF
SDDDIPGRHSLKDSLSAVRNAVQAAARIHEVFRVQSFQRKQLIEFGDDKFEMSDEQALSL
ISVKSNRTVQNDEPIHTAAVRIQNKFRGWKGRKEFLIIRQRIVKIQALVRGHQVRKHYRK
IVWSVGIVEKVILRWRRKGSGLRGFRPGLIEGTMQSASSKEDDYDFLKEGRKQTEARLDK
ALARVRSMVQYPEARDQYRRLLNGVTEFQETKVVLEKLLNSSEEAMEGDDMIDIEMLLGD
DAFTPTVS
>PbCAMTA7
MAEGRRYGLSTQLDIEQILLEAQNRWLRPPEICEILRNYQKFRIAPEPPNRPPSGSLFLF
DRKVQRYFRKDGHNWRKKKDGKTVKEAHERLKAGSVDVLHCYYAHGEGNENFQRRSYWML
EEDLMHIVLVHYREVKGNKLNYRTRDPDETSQSTHSGSPIRSNLFNDNNQVLSRTMDTSS
LNSAHTSELDDAESDNHQASSRYHSFLDLQQSEDRPGINSQMGVGLLNSYFPASYPTDQS
DYQGKQLNIPGLNFVSLGQENLTTKINNSGLGLRFHESRKQNGAASWEEALEHSATQFQG
APFGLPVSSSSPSNVMGIHHPENVIIGQLSTEDYVVKQEVVSSLKGQLQWQIASEDDSSH
VAAWPMDQKLHTELVSDLSTGFQEQKGHYVDVDNDPDTFSANSDQGNESFMPNEFHLNLS
NAEVGGLLTSDLDTEKDQVKEEDLSHYSSTRHPLIEDDVFKKFDSFSRWMSKELGEVHEA
RVPPSSGIYWSTVESGNAIEASGMSPQVHDAYLLSPPSLSQDQLFSILDFSPNWAYTGME
TKVLITGTFLKDKNDAMKCKWSCMFGEVEVLAEVLADGVLRCRAPVHAAGRVPFYITCSN
RVACSEVREFEYRIGHSCHMDTDTYGGSTTEMILRVRLGKLLSLDSVGYANSLSNSAGEK
SHISSKISSLLKEDDNEWFQMEKLTTDDEFSLGEVKERLLQRILKDKLHAWLLHKVADDG

KGPSVLDKEGQGVLHLAAALGYDWAIAPTIAAGVNINFRDVNGWTALHWAASCGREQTVI
TLVALGGAPGALTHPTPEFPSGRAPADLASINGHKGIAGYLAESSLKAHLEALTLETFED
GNASGVPRVKAIQTIAELSPTQYIDGDIDGQPLKDSLTAVRNAVQAAARIHEVFRVQSFH
RKQLIESGDDKFGMSDERALSFISFKSNRAGQHNEPTHTAAIRIQNKFRSWKGRKEFLIF
RQRIVKIQAHVRGYQVRKHYRKIIWSVGILEKVILRWRRKGSGLRGFRPEGLIEGQSRQG
TSSKEDDYDFLKEGRKQTEARLQKALARVRSMVQYPDARDQYHRLLNGVSEFQETKVVLE
KLLNDSEEVVEGDDMIDFEALLGDDTFMSTAT
>PbCAMTA8
MADGRRYAFSPQLDIEQILLEAKNRWLRPSEICEILRNYQRFQLTPDPPNRPPGGSLFLF
DRKALRYFRKDGHRWRKKKDGKTVREAHEKLKAGSVDVLHCYYAHGEDNENFQRRSYWML
DGQLEHIVLVHYREVKEGSRSGIPRLLNTDPEPLNHCQSTQTSSAACSHVQVQLLLQRRH
RMHQAQIASLHPNDTAERNQLGCSVAVSSLYSRGLTDATGIIGGQINPYFVNQGPTLNLL
LNGGLHADYRTTQETVGPYGLFKGGEDGRMFPCAHPQTSTKNSLQENYKEDEQDFHANTQ
FQSNWSINVPESSLYNEHGELKKLDSFGRWMNNEIGKDCDDSLMASDSCNYWNTLDTQND
DKEVSSLSRQMQLDVDSLGPSLSQVQLFSILDFSPDWAFSGFETKVLISGTFLGGMENRN
TARWCCMFGEVEVPAEVVANNALRCHAPSHVPGRVPFYITCSNRLACSEVREFEYRDNPS
ETISILGVKGESEEELCLQIRFTKMILRGVDRKWLDCSLEKCEKCNLKKDTYSMGNAAKA
MDKNHENPRKALMQKLLKDRLYEWLVCKAHEGGKGPNILDDKGQGVIHLAAALGYEWAMN
PIVAAGISPSFRDSRGWTALHWAAFFGREGTIVALVRLGAAPGAVEDPTSKFPEGRTAAD
LASSRGHKGIAGYLAEADLTSHPSSLTLNENVRDNVLDNVSVNLDAEKAIETLVEQSVGP
LDVDQEDCLSLRNSLAAVRNSAQAAARIQLHCVFVHSVRGSYQKGSHFSDSLHTAAVRIQ
QKYRGWKGRREFLKIRNRIVKIQAHVRGHQVRKQYKKVVWSVSIVEKAILRWRRKGAGLR
GFQTGKTDEYEFLRVGRKQKVAGVEKALARVQSMVRYREGRDQYMRLVTNFQKLQMGSEG
ASGKTQISEESEIDANHHQIHFYLDTPTLRSWICSLAFSFLCCVKLVADGFGASLNYCVK
LVTDAGEFNDTNYERSGHVNFV
>PbCAMTA9
MKPKKSSNRRSKKNPSNSQTKMVTKSTNSASKSDTIDSLISDGDQQSLDVSQGTEMGPSE
SSRISGGRGKRPSSLATAPGAASPATFPTSEEEEVADILLLLKGGDGDASDRDEKEQELS
LLIQKGLSQNEHELDIISKNFNDRPKKVSCRDKFYEQIEKGDYTVGPYRFFKGGEDGRMF
PCAHPQMSTKNSLQMQLDVDSLGPSPSQVQLFSILDVSPDWAFSGFETKVLILGTFLGGM
ENHNTTRWCCMFGEVEVPAEVLGTTALRCHAPVREFEYRDNPSETISSLGVNGESEEELC
LQIRLTKMILRGVDSKWLDCSLEKCEKCNLKKDMYSMGTADENDWGKIEISVNAMGKNHE
NRRKALMQKLLKERLFEWLVCKAHEGGKGPNILDDEGQGVIHLAAALGYEWAMSPIVAAG
ICPSFRDSRGWTGLHWAAFFGRGNSSCTSRLGAAPGAVEDPTSKFPEGRTAADLASSRGH
KGIAGYLAEADLTRLKGNVLDSVSVNLAAEKAIETPVEQSVVGPLDALSRRK
>PbCAMTA10
MAGKEDEKLPSENKGSKSEESSGEPQAGQRRTTSGTGFPANPFDFSAMSGLLNDPSIKEL
ADQIAKDPAFNQMAEQLQKSVQGAGQDGIPQLDTQQYFSTMQQVMQNPQFMTMAERLGNA
LMQDPSMSGMLDSLANPSHKEQLEERMARIKEDPSLKPILDEIESGGPAAMMRYWNDPEV
LQKLGQAMGLGAAGEAATSAELSGQDEAEEETGYEDESTVHHTASVGDVEGLKSALAAGA
DKDEEDSEGRTALHFACGYGEVKCAQVLLEAGAAVDALDKNKNTALHYAAGYGRKECVAL

LLDNGAAVTLQNLDGKTPIDVAKLNNQQEVLKLLEKDAFV
>PbCAMTA11
MDRLISLEPSNLVTVRVEPGQRCYGELTLRNVMYTMPVAFRLQPINKGRYTVRPQSGIIS
PLATLTVEIIYNMLPSSTLPDSAPHSDDSFLLHSVVVPGAAVKDPSSTLDLQVFIDSGIK
ILFVGSAILSRLVAVGSMDEVREVLERSDSAWHPADSVDSQGQTLLHIAIAASRADLVQL
LLEFEPNVEARSRAGRSPLEAAAAAGEALIVELLLAHHASTDRSSSSAWGPLHFAAGAGH
LEVMRLLLLKGADLDAPTSDGPAPGLHPLLLACGAHVNVHGTDDGDTPLHIAAGLGDEHM
VKLLLQKGANKDIRNRLGKTAYDVAAEGGHNRLFDALRLGDSLCAAARKGELRTVQRLLE
NGALINGRDQHGWTALHRVSFKGRVDIARMLLSKGLDVNARDEDGYTALHCATEAGQAEI
IELLVKKGADVDARTNKGVTALQIADSLHYSGIARILVQGGAIRDGAAPPVGRPLIPFFG
APTREQEKRLKKKVNRKSVRRSSFDRALPLACST
>PbCAMTA12
MSSLGGAVASSSPRSPFHLSPRSPFHLLPPFSSAARSSSSSSSSFSLRAPKSSSPFFSSG
VGVSLRSKSTTPKISNWVASKRVPLQTQESNWEYPYDGSDSDYDDDDEQGNKEEREQEFE
SHFEKDEEEKRIEAGVAAARYVNTATAPTEYEEEIIKEVELLLDPEEKAILEQNEYPVLS
KISTTKWSPFHSLALSVQIPFMDKLLQSSFDIDAVDKDGFTALHKAVIGKKEAVISHLLR
KGANPHVRDKDGVTPLHYAVQVGAIQTVKLLIKYKVDVNVADNEGWTPLHVAVQSRSRDI
AKVLLVNGADRTRRNKDGKAPLDLALCFGKDFKSYELSKLLKLVPANRDL
>PbCAMTA13
MSKKEKRAILQQLEFGRKVERKLNPVVLPNMADYSSYRSLKLSQLNPVVLDNPGQLKTEK
RREEATGSESSDRDVGELDGGALGRDSSSRVVPRNPRLRVGGRGLEDVSEFFNSSDYNPE
EKEKSHEGPRRLFTKEEKALLNKRIPNLADATSTIWLPLHTLVASGEFYLVDKLRKHNVD
FNATDKDGLTALHRAIICKKQAVTNYLLRESANPFVRDKEGATLMHYAVLSASSPAIKLL
LLYNVDINLSDDDGWTPLHLAVQTQRTDVVRLLLIKGADKTLKNQDGLTPLDLCLYSGRS
VRTYELIKLLKVLPNPQ
>PbCAMTA14
MASPDSEIEVVSDSQNHQNTNSLNGFPAEDIIVDVYSASAYGSLPKLRKFVEVDGFPLSQ
PDGNGYYALQWAALNNYADIAQYIIERGGEVNAIDNSQQTALHWAAVRGSVAAADVLLQN
GARIEAADVNGYRAVHVAAQYGQTGFLNHIVTRYGADFDAPDNDGRSPLHWAACKGFADT
IRLLLFRDADQRRQDKDGCTPVHWAAIRGNVEACTVLVHAGTKHELLVKDNAGFTPVQLA
SDKGHRHIAFFLSNARRTLGNSGEDKVFIGKIGNIGYAPILLFIMIAEMVLFMNSVLTGH
CGCWTLGLDCCFFAVVTILMFYRCSSKDPGYIKKSSGQADVDDPLLNIDLNSSSIWTGNW
SQLCPTCKIIRPMRSKHCPSCKHCVEQFDHHCPWISNCVGKKNKWDFFVFLCLATLSATI
AAAVAVQRMWTESPVLPSAGKWIHFMVTEHPGIILFLVMDVICLSAAVALTIAQASQIAR
NITTNELANASRYGYLRGPEGKFRNPYNHGCRKNCTDFLIHGYSNDDEIAWPSLQQATN
>PbCAMTA15
MASEIEIVEDGGGDVSTAQEDEIALPNDVYTAAAHGDLEKLQRLVESEGCSFSETDSLGY
YALQWAALNNRTAAAQYIIEHGGDVNAADHTGQTALHWSAVRGAIQVAELLLQEGARVSA
TDLYGYQATHVAAQYGQTAFLYHIVTKWNADPDVPDNDGRGPLHWAAYKGFADCIRLLLF
LDAYRGRQDKEGCTPLHWAAIKGNLEACTVLVQAGKKEDLVVTDNTGFTPAQLASDKNHR
QVAFFLGNARRLLDKRCDGNSWLGKFSKLGLAPVLWCMIIILLSIYVHSVVTASDLLKLT

ALFELFAWLGFFLATAGLVMFYRCSRKDPGYIKMNVRDSQNLKDDEPLLKLDLNNPALLA
GNWSQLCATCKIVRPLRAKHCSTCDRCVEQFDHHCPWVSNCIGKKNKWDFFMFLILEISA
MVITGAIAATRILMDPTAPSTFRAWMNHIVTHHMGSISFLIMDFLLFFGVAVLTVVQASQ
ISHNITTNEMANAMRYSYLRGPSGGYRNPYDHGIQKNCSDFLIKGYNEDIERVEQQAHSE
EIGMIRMTRNANSQNRDNNSRHTNSNGHVSVDVDSESTRSQGHKHSSHCNHNHGKTDGFP
LGLGLGLGRNNIHHHHTRSVVAS

>PbCAMTA16

MVSEIEIVEDGGARDQEAASSAGDAAGEEVLRNDVFTAAAYGDLEKLQRLVESEGCSVSE
PDSLGYYALQWAALNNRTAAAQYIIEHGGDVNAADHTGQTALHWSSVRGAIQVAELLLQE
GARVSATDLYGYQTTHVAAQYGQTAFLYHIVTKWNADPDIPDNDGRSPLHWAAYKGFSDC
IRLLLFLDAYRGRQDKEGCTPLHWAAIRGNLEACTVLVQAGKKEDLMVTDNTGFTPAQLA
SDKNHRQVAFFLGNARRLLDKRCDGNSRLGKLSKLGLAPVLWCMIIFLLIIYIHTVITAS
QLLKLTAAFGLFAWLGVFLATSGLVMFYRCSRKDPGYIKMNVHDSQNLKDDEPLLRSELN
NPVLLAGNWSQLCATCKIVRPLRAKHCSTCDRCVEQFDHHCPWVSNCIGKKNKRDFFMFL
ILEVSAMLVTGAVAAIRILTDPTAPSSFGEWVNHAGTHHIGAISFLIMDFLLFFGAAVLT
FIQASQISRNITTNEMANAMRYSYLRAPTGGFRNPYDRGFKKNCSDFLINGYNEDIERAE
EPVCAEGMGMIPMTRNTNLQNGDHHSHHTNHSHHTNGNGHVCVDVDSKNTRPQAHKHSSN
SNHNHLGKSDGFPLGLGLGLGRSNLHQHHTRAVPP

>PbCAMTA17

EDEIALPNDVYTAAAHGDLEKLQRLVESEGCSFSETDSLGYYALQWAALNNRTAAAQYII
EHGGDVNAADHTGQTALHWSAVRGAIQVAELLLQEGARVSATDLYGYQATHVAAQYGQTA
FLYHIVTKWNADPDVPDNDGRGPLHWAAYKGFADCIRLLLFLDAYRGRQDKEGCTPLHWA
AIKGNLEACTVLVQAGKKEDLVVTDNTGFTPAQLASDKNHRQVAFFLGNARRLLDKRCDG
NSWLGKFSKLGLAPVLWCMIIILLSIYVHSVVTASDLLKLTALFELFAWLGFFLATAGLV
MFYRCSRKDPGYIKMNVRDSQNLKDDEPLLKLDLNNPALLAGNWSQLCATCKIVRPLRAK
HCSTCDRCVEQFDHHCPWVSNCIGKKNKWDFFMFLILEISAMVITGAIAATRILMDPTAP
STFRAWMNHIVTHHMGSISFLIMDFLLFFGVAVLTVVQASQISHNITTMKWQMQCAIVIS
EALVVGTEIRMIMAFKNCSDFLIKGYNEDIERVEQQAHSEEIGMIRMTRNANSQNRDNNS
RHTNSNGHVSVDVDSESTRSQGHKHSSHCIITMVRLMGSLWAWGLGSDETIFITIIHVLL

>AtCAMTA1

MVDRRSFGSITPPLQLDMEQLLSEAQHRWLRPTEICEILQNYHKFHIASESPTRPASGSL
FLFDRKVLRYFRKDGHNWRKKKDGKTIREAHEKLKVGSIDVLHCYYAHGEANENFQRRCY
WMLEQHLMHIVFVHYLEVKGNRTSIGMKENNSNSVNGTASVNIDSTASPTSTLSSLCEDA
DTGDSQQASSVLRPSPEPQTGNRYGWTPAPGMRNVSQVHGNRVRESDSQRLVDVRALDTV
GNSLTRFHDQPYCNNLLTQMQPSNTDSMLVEENSEKGGRLKAEHIRNPLQTQFNWQDDTD
LALFEQSAQDNFETFSSLLGSENLQPFGISYQAPPSNMDSEYMPVMKILRRSEDSLKKVD
SFSKWAIKELGEMEDLQMQSSRGDIAWTTVECETAAAGISLSPSLSEDQRFTIVDFWPKS
AKTDAEVEVMVIGTFLLSPQEVTKYNWSCMFGEVEVPAEILVDGVLCCHAPPHTAGHVPF
YVTCSNRFACSEVREFDFLSGSTQKINATDVYGTYTNEASLQLRFEKMLAHRDFVHEHHI
FEDVGDKRRQISKIMLLKEEKEYLLPGTYQRDSTKQEPKGQLFRELFEEELYIWLIHKVT
EEGKGPNILDEDGQGILHFVAALGYDWAIKPVLAAGVNINFRDANGWSALHWAAFSGREE

TVAVLVSLGADAGALTDPSPELPLGKTAADLAYANGHRGISGFLAESSLTSYLEKLTVDS
KENSPANSCGEKAVQTVSERTAAPMTYGDVPEKLSLKDSLTAVRNATQAADRLHQVFRMQ
SFQRKQLCDIGDDEKIDISDQLAVSFAASKTKNPGQGDVSLSCAATHIQKKYRGWKKRKE
FLLIRQRIVKIQAHVRGHQVRKQYRTVIWSVGLLEKIILRWRRKGNGLRGFKRNAVAKTV
EPEPPVSAICPRIPQEDEYDYLKEGRKQTEERLQKALTRVKSMVQYPEARDQYRRLLTVV
EGFRENEASSSASINNKEEEAVNCEEDDFIDIESLLNDDTLMMSISP
>AtCAMTA5
MAGVDSGKLIGSEIHGFHTLQDLDIQTMLDEAYSRWLRPNEIHALLCNHKFFTINVKPVN
LPKSGTIVLFDRKMLRNFRKDGHNWKKKKDGKTIKEAHEHLKVGNEERIHVYYAHGEDTP
TFVRRCYWLLDKSQEHIVLVHYRETHEVHAAPATPGNSYSSSITDHLSPKIVAEDTSSGV
HNTCNTGFEVRSNSLGSRNHEIRLHEINTLDWDELLVPADISNQSHPTEEDMLYFTEQLQ
TAPRGSVKQGNHLAGYNGSVDIPSFPGLEDPVYQNNNSCGAGEFSSQHSHCGVDPNLQRR
DFSATVTDQPGDALLNNGYGSQDSFGRWVNNFISDSPGSVDDPSLEAVYTPGQDSSTPPT
VFHSHSDIPEQVFNITDVSPAWAYSTEKTKILVTGFFHDSFQHLGRSNLICICGELRVPA
EFLQMGVYRCFLPPQSPGVVNLYLSVDGNKPISQLFSFEHRSVQFIEKAIPQDDQLYKWE
EFEFQVRLAHLLFTSSNKISVLTSKISPENLLEAKKLASRTSHLLNSWAYLMKSIQANEV
PFDQARDHLFELTLKNRLKEWLLEKVIENRNTKEYDSKGLGVIHLCAVLGYTWSILLFSW
ANISLDFRDKQGWTALHWAAYYGREKMVAALLSAGARPNLVTDPTKEFLGGCTAADLAQQ
KGYDGLAAFLAEKCLVAQFKDMQTAGNISGNLETIKAEKSSNPGNANEEEQSLKDTLAAY
RTAAEAAARIQGAFREHELKVRSSAVRFASKEEEAKNIIAAMKIQHAFRNFEVRRKIAAA
ARIQYRFQTWKMRREFLNMRKKAIRIQAAFRGFQVRRQYQKITWSVGVLEKAILRWRLKR
KGFRGLQVSQPDEKEGSEAVEDFYKTSQKQAEERLERSVVKVQAMFRSKKAQQDYRRMKL
AHEEAQLEYDGMQELDQMATEES
>AtCAMTA3
MAEARRFSPVHELDVGQILSEARHRWLRPPEICEILQNYQRFQISTEPPTTPSSGSVFMF
DRKVLRYFRKDGHNWRKKKDGKTVKEAHERLKAGSVDVLHCYYAHGQDNENFQRRSYWLL
QEELSHIVFVHYLEVKGSRVSTSFNRMQRTEDAARSPQETGDALTSEHDGYASCSFNQND
HSNHSQTTDSASVNGFHSPELEDAESAYNQHGSSTAYSHQELQQPATGGNLTGFDPYYQI
SLTPRDSYQKELRTIPVTDSSIMVDKSKTINSPGVTNGLKNRKSIDSQTWEEILGNCGSG
VEALPLQPNSEHEVLDQILESSFTMQDFASLQESMVKSQNQELNSGLTSDRTVWFQGQDM
ELNAISNLASNEKAPYLSTMKQHLLHGALGEEGLKKMDSFNRWMSKELGDVGVIADANES
FTQSSSRTYWEEVESEDGSNGHNSRRDMDGYVMSPSLSKEQLFSINDFSPSWAYVGCEVV
VFVTGKFLKTREETEIGEWSCMFGQTEVPADVISNGILQCVAPMHEAGRVPFYVTCSNRL
ACSEVREFEYKVAESQVFDREADDESTIDILEARFVKLLCSKSENTSPVSGNDSDLSQLS
EKISLLLFENDDQLDQMLMNEISQENMKNNLLQEFLKESLHSWLLQKIAEGGKGPSVLDE
GGQGVLHFAASLGYNWALEPTIIAGVSVDFRDVNGWTALHWAAFFGRERIIGSLIALGAA
PGTLTDPNPDFPSGSTPSDLAYANGHKGIAGYLSEYALRAHVSLLSLNDKNAETVEMAPS
PSSSSLTDSLTAVRNATQAAARIHQVFRAQSFQKKQLKEFGDKKLGMSEERALSMLAPKT
HKSGRAHSDDSVQAAAIRIQNKFRGYKGRKDYLITRQRIIKIQAHVRGYQFRKNYRKIIW
SVGVLEKVILRWRRKGAGLRGFKSEALVEKMQDGTEKEEDDDFFKQGRKQTEDRLQKALA
RVKSMVQYPEARDQYRRLLNVVNDIQESKVEKALENSEATCFDDDDDLIDIEALLEDDDT

LMLPMSSSLWTS
>AtCAMTA2
MADRGSFGFAPRLDIKQLLSEAQHRWLRPAEICEILRNHQKFHIASEPPNRPPSGSLFLF
DRKVLRYFRKDGHNWRKKKDGKTVKEAHEKLKVGSIDVLHCYYAHGEDNENFQRRCYWML
EQDLMHIVFVHYLEVKGNRMSTSGTKENHSNSLSGTGSVNVDSTATRSSILSPLCEDADS
GDSRQASSSLQQNPEPQTVVPQIMHHQNASTINSYNTTSVLGNRDGWTSAHGNRVKGSNS
QRSGDVPAWDASFENSLARYQNLPYNAPLTQTQPSTFGLIPMEGKTEKGSLLTSEHLRNP
LQSQVNWQTPVQESVPLQKWPMDSHSGMTDATDLALFGQGAHENFGTFSSLLGSQDQQSS
SFQAPFTNNEAAYIPKLGPEDLIYEASANQTLPLRKALLKKEDSLKKVDSFSRWVSKELG
EMEDLQMQSSSGGIAWTSVECENAAAGSSLSPSLSEDQRFTMIDFWPKWTQTDSEVEVMV
IGTFLLSPQEVTSYSWSCMFGEVEVPADILVDGVLCCHAPPHEVGRVPFYITCSDRFSCS
EVREFDFLPGSTRKLNATDIYGANTIETSLHLRFENLLALRCSVQEHHIFENVGEKRRKI
SKIMLLKDEKEPPLPGTIEKDLTELEAKERLIREEFEDKLYLWLIHKVTEEGKGPNILDE
DGQGVLHLAAALGYDWAIKPILAAGVSINFRDANGWSALHWAAFSGREDTVAVLVSLGAD
AGALADPSPEHPLGKTAADLAYGNGHRGISGFLAESSLTSYLEKLTVDAKENSSADSSGA
KAVLTVAERTATPMSYGDVPETLSMKDSLTAVLNATQAADRLHQVFRMQSFQRKQLSELG
GDNKFDISDELAVSFAAAKTKKSGHSSGAVHAAAVQIQKKYRGWKKRKEFLLIRQRIVKI
QAHVRGHQVRKQYRAIIWSVGLLEKIILRWRRKGSGLRGFKRDTISKPTEPVCPAPQEDD
YDFLKEGRKQTEERLQKALTRVKSMAQYPEARAQYRRLLTVVEGFRENEASSSSALKNNT
EEAANYNEEDDLIDIDSLLDDDTFMSLAFE
>AtCAMTA6
MDGDGLGRLIGSEIHGFHTLQDLDVQTMLEEAKSRWLRPNEIHAILCGRIILFDRKMLRN
FRKDGHNWKKKKDGRTVKEAHEHLKVGNEERIHVYYAHGEDNTTFVRRCYWLLDKARENI
VLVHYRDTQEAATTSGDSISSPISVSEQTFPNRVAAEDIDTVVRNHDISLHDINTLDWDE
LLVPTDLNNQSAPTVDNLSYFTEPLQNAANGTAEHGNATVADGSLDALLNDGPQSRESFG
RWMNSFISESNGSLEDPSFEPMVMPRQDPLAPQAVFHSHSNIPEQVFNITDVSPAWAYSS
EKTKILVTGFLHDSYQHLERSNLYCVCGDFCVPAEYLQAGVYRCIIPPHSPGMVNLYLSA
DGHKPISQCFRFEHRAVPVLDKTVPEDNQDSKWEEFEFQVRLSHLLFTSSNKLNVLSSKI
SPHNLRDAKKLASKTNHLLNSWAYLVKSIQGNKVSFDQAKDHLFELSLKNRLKEWLMEKV
LEGRNTLDYDSKGLGVIHLCASLGYTWSVQLFSLSGLSLNFRDKQGWTALHWAAYYGREK
MVAALLSAGARPNLVTDSTKDNLGGCMAADLAQQNGYDGLAAYLAEKCLVAQFRDMKIAG
NITGDLEACKAEMLNQGTLPEDEQSLKDALAAYRTAAEAAARIQGAFREKALKAARSSVI
QFANKEEEAKSIIAAMKIQNAFRKYDTRRKIEAAYRIQCRFQTWKIRREYLNMRRQAIRI
QAAFRGLQARRQYKKILWSVGVLEKAVLRWRQKRKGFRGLQVAAEEDSPGEAQEDFYKTS
QRQAEERLERSVVRVQAMFRSKKAQQDYRRMKLTHEEAQVNHLTFLNLSFGKKNSNRR
>AtCAMTA4
MSSVAEDNSFTCDIATIFVAICRNPPANPSDSLFQYEISTLYQEAHSRWLKPPEVLFILQ
NHESLTLTNTAPQRPTSGSLLLFNKRVLKFFRKDGHQWRRKRDGRAIAEAHERLKVGNAE
ALNCYYAHGEQDPTFRRRIYWMLDPEYEHIVLVHYRDVSEREEGQQTGGQVYQFAPILST
QNVSYNQYIGDSSDIYQQSSTSPGVAEVNSNLEGSASSSEFGQALKMLKEQLSIGDEHVN
SVDPHYIQPESLDSLQFLEYSDIDHLAQPTTVYQRPENNKLERCYGGNFGAQYSAKNDSN

KLERCYGGYVGGAEYHSSNLMLVKNGSGPSGGTGGSGDQGSESWKDVLEACEASIPLNSE
GSTPSSAKGLLAGLQEDSNWSYSNQVDQSTFLLPQDLGSFQLPASYSALVAPENNGEYCG
MMEDGMKIGLPFEQEMRVTGAHNQKFTIQDISPDWGYANETTKVIIIGSFLCDPTESTWS
CMFGNAQVPFEIIKEGVIRCEAPQCGPGKVNLCITSGDGLLCSEIREFEYREKPDTCCPK
CSEPQTSDMSTSPNELILLVRFVQTLLSDRSSERKSNLESGNDKLLTKLKADDDQWRHVI
GTIIDGSASSTSTVDWLLQELLKDKLDTWLSSRSCDEDYITCSLSKQEQGIIHMVAGLGF
EWAFYPILAHGVNVDFRDIKGWSALHWAAQFGSEKMVAALIASGASAGAVTDPSRQDPNG
KTAASIAASNGHKGLAGYLSEVALTNHLSSLTLEETENSKDTAQVQTEKTLNSISEQSPS
GNEDQVSLKDTLAAVRNAAQAAARIQAAFRAHSFRKRKQREAALVACLQEYGMYCEDIEG
ISAMSKLTFGKGRNYNSAALSIQKNFRGYKDRKCFLELRQKVVKIQAHVRGYQIRKNYKV
ICWAVRILDKVVLRWRRKGVGLRGFRQDVESTEDSEDEDILKVFRKQKVDVAVNEAFSRV
LSMSNSPEARQQYHRVLKRYCQTKAELGKTETLVGEDDDGLFDIADMEYDTLFSLP
>TaCAMTA1a-A
MAEMHKYGLSNQPPDIPQILQEAQNRWLRPTEICQILSNYKKFSIAPEPPNRPPSGSLFL
FDRKILRYFRKDGHIWRKKKDGKTVKEAHEKLKVGSVDVLHCYYAHGEENENFQRRTYWL
LEEGFMNIVLVHYLEIKGGKQSFSRSKEAEDSPACSNSFASQSQVASQTMDAESPYSGQI
SEYEDAETDNSRASSRYHPFVEMQQPVDGVMMDNRLGAPAPSTSVNNLGYQDENQASTAN
ISNNFVTHHGIASVFNDVGAGLRSGSKTALDSVHFGEPFPEYPTGFTEPTLYSSVTTMGS
NNLDDNSRLETLMTEALYTNNLTQKETDALSAAGMTSSQVHNDSYTDGSMGYPLLKQSSL
DLFKIEPNGLKKFDSFSKWMSDELAADLDIKSTSDAFWSSTETVNVADGSSMSIPMNEQL
DAYVVSPSLSQDQLFSIIDVSPSWAYTGSQNKVLITGTFLTNKEHVENCKWSCMFGDVEV
PVEVLADGSLRCYTPVHQSGRVPFYVTCSNRVACSEVREFEFHDSETQHMEAADPHITGI
NEMHLHIRLEKLLSLGPDDYEKYVMSGGNEKSELISTIGSLMLDDKFTNLSAPSDEEFSA
AQDKNLEKSVKDKLYYWLIHKIHDDGKGPNVLGKEGQGVIHLVAALGYDWAIRPIIAAGV
HVNFRDVRGWTALHWAASCGRERTVGALITNGAAAGALTDPTPHFLSGRTPADLASDNGH
KGIAGFLAESALTSHLSALTLKEAKGCNVEEICGSIEADGFAEPSSAQLSRQDSQAESLK
DSLSAVRKSTLAASKIFQAFRVESFHRKKVVEYGDDDCGLSDERTLSLVSLKNTKSGQND
MPHSAAVRIQNKFRGWKGRKEFMIIRQKIIKIQAHIRGHQVRRNYKKVVWSVGIVEKVIL
RWRRKGRGLRGFQPDKQLEGPSSQIQPAEGGSAEGEDEYDFLKDGRKQAEGRLQRSLARV
KSMTQYPEAREQYSRLQACVTELQESKAIQDKMLSDAAGVGGGDFMVDLEDLCADELLDT
PMSNIL
>TaCAMTA1a-B
MAEMHKYGLSNQPPDIPQILQEAQNRWLRPTEICQILSNYKKFSIAPEPPNRPPSGSLFL
FDRKILRYFRKDGHIWRKKKDGKTVKEAHEKLKVGSVDVLHCYYAHGEENENFQRRTYWL
LEEGFMNIVLVHYLEIKGGKQSLSRSKEAEDSPACSNSFASQSQVASQTMDAESPYSGQI
SEYEDAETDNSRASSRYHPFVEMQQPVDGVMMDNRLGAPAPSTSVNNLGYQDEKQARTAN
ISNNFVTHHGITSVFNDVGAGLRSGSKTALDSVHFGEPFPEYPTGFMEPTLYSSVTTMGS
NSLDDNSRLETLMTEALYTNNLTQKETDALSAAGMTSSQVHNDSYTDGSMGYPLLKQSSL
DLFKIEPNGLKKFDSFSKWMSDELAADLDIKSSSDAFWSSTETVNVADGSSMSIPMNEQL
DAYAVSPSLSQDQLFSIIDVSPSWAYNGSQNKVLITGTFLTNKEHVENCKWSCMFGDVEV
PVEVLADGSLRCYTPVHQSGRVPFYVTCSNRVACSEVREFEFRDSETQYMEAADPHITGI

NEMHLHIRLEKLLSLGPDDYEKYVMSDGNEKSELISTIGSLMLDDKFTNLSAPSDEKELS
AAQDKNLERSVKDKLYYWLIHKIHDDGKGPNVLGKEGQGVIHLVAALGYDWAIRPIIAAG
VHVNFRDVRGWTALHWAASCGRERTVGALITNGAAAGALTDPTPHFLSGRTPADLASENG
HKGIAGFLAESALTSHLSALTLKEAKGCNVEEICGSAEADGFAEPSSAQLSRQDSQAESL
KDSLSAVRKSTLAASKIFQAFRVESFHRKKVVEYGDDDCGLSDERTLSLVSLKNTKSGQN
DMPHSAAVRIQNKFRGWKGRKEFMIIRQKIIKIQAHVRGHQVRRNYKKVVWSVGIVEKVI
LRWRRKGRGLRGFQPDKQLEGPSSQIQPAEGGSAEGEDEYDFLKDGRKQAEGRLQRSLAR
VKSMTQYPEAREQYSRLQACVTELQESKAIQDKMLSDAAGVDGGDFMVDLENLCADELLD
TPMSTVL
>TaCAMTA1a-D
MAEMHKYGLSNQPPDIPQILQEAQNRWLRPTEICQILSNYKKFSIAPEPPNRPPSGSLFL
FDRKILRYFRKDGHIWRKKKDGKTVKEAHEKLKVGSVDVLHCYYAHGEENENFQRRTYWL
LEEGFMNIVLVHYLEIKGGKQSFSRSKEAEDSPACSNSFASQSQVASQTMDAESPYSGQI
SEYEDAETDNSRASSRYHPFVEMQQPVDGVMMDNRLGAPAPSTSVNNLGYQDENQARTAN
ISNNFVTHHGIASVFNDVGAGLRSGSKTALDSVHFGEPFPEYPTGFTEPTLYSSVTTMGS
NNLDDNSRLETLMTEALYTNNLTQKETDALSAAGMTSSQVHNDSYTDGSMGYPLLKQSSL
DLFKIEPNGLKKFDSFSKWMSDELAADLDIKSTSDAFWSSTETVNVADGSSMSIPMNEQL
DAYVVSPSLSQDQLFSIIDVSPSWAYTGSQNKVLITGTFLTNKEHVENCKWSCMFGDVEV
PVEVLADGSLRCYTPVHQSGRVPFYVTCSNRVACSEVREFEFHDSETQHMEAADPHITGI
NEMHLHIRLEKLLSLGPDDYEKYVMSGGNEKSELISTIGSLMLDDKFTNLSAPSDEEFSA
AQDKNLEKSVKDKLYYWLIHKIHDDGKGPNVLGKEGQGVIHLVAALGYDWAIRPIIAAGV
HVNFRDVRGWTALHWAASCGRERTVGALITNGAAAGALTDPTPHFLSGRTPADLASDNGH
KGIAGFLAESALTSHLSALTLKEAKGCNVEEICGSAEADGFAEPSSAQLSRQDSQAESLK
DSLSAVRKSTLAASKIFQAFRVESFHRKKVVEYGDDDCGLSDERTLSLVSLKNTKSGQND
MPHSAAVRIQNKFRGWKGRKEFMIIRQKIIKIQAHIRGHQVRRNYKKVIWSVGIVEKVIL
RWRRKGRGLRGFQPDKQLEGPSSQIQPAEGGSAEAEDEYDFLKDGRKQAEGRLQRSLARV
KSMTQYPEAREQYSRLQACVTELQESKAIQDKMLSDAAGGDGGDFMVDLEDLCADELLDT
PMSTIL
>TaCAMTA1b-A
MAEGRRYGIAPQLDMEQILKEAQTRWLRPTEICEILKNYRNFRIAPEPPNMPASGSLFLF
DRKVLRFFRKDGHNWRKKKDGKTVKEAHERLKSGSIDVLHCYYAHGEENINFQRRSYWML
EEDYMHIVLVHYLEVKAGKSSSRTRGHDNMLQGAYVDSPLSHLPSQSTDGESSLSGRASE
YEAESADIYSGGAGYHSISRMQQHENGGGSIIDASVVSSYSPASSVGNHQGLQATSPNTG
FYSHYQDNSPVIHNESTFGITFNGPSTQFDLSSWNEMTKLNKEIHQLPPYQSHVPSEQPP
FTEGPGIESFSFDEVYSNGLDIKDDGHADTDREALWQLPSANDGTTTEFLQLPSAIDGRT
TEFQLPSATDSTFATVDNFEQHSKLLEEAINFPVLKTQSSNLSDILKNSFKKSDSFTRWM
SKELAEVDDSQVKFSSGLYWNSEDADNIIGASSRDQLDQFTLDPMVAQDQLFSITEYFPS
WTYAGSKTRVLVTGRFLTSDEVIKLKWSCMFGEVEVPADILADGTLRCYSPSHKPGRVPF
YVTCSNRLACSEVREFEYRPSDSQYMDAPSPHGATNKIYLQARLDELLSLGRDQQDEFQA
ALSNPTKELIDLNKKITSLMTNNDQWSELLKFADDNQLAPDDRQDQFVESGIKEKLHIWL
LHKAGGGGKGPSVLDDEGQGILHLAAALGYDWAIRPTITAGVSINFRDVHGWTALHWAAF

CGRERTVVALIALGAAPGALTDPRPDFPSGRTPADLASFNGHKGISGFLAEFSLTSHLQT
LNLKEAMGSNASEISGLPGIGDVTGRTASPSAGQGLQAGSMGDSLGAVRNAAQAAARIYQ
VFRVQSFQRKQAVQYEDDNGVISDERAMSLLSYKPSKPGQFDPMHAAATRIQNKFRGWKG
RKEFLLIRQRIVKIQAHVRGHQVRKHYRKIIWSVGIVEKVILRWRRRGAGLRGFRSTEVA
TDSSTSSSSVDVIPVKPAEDDYNFLQEGRKQTEERLQRALARVKSMVQYPEARDQYQRIM
TVVTKMQESQPVEESMLEESTEMDEGFLMSEFKELWDDDMPLPGYF
>TaCAMTA1b-B.1
MAEGRRYGIAPQLDMEQILKEAQTRWLRPTEICEILKNYRNFRIAPEPPNMPASGSLFLF
DRKVLRFFRKDGHNWRKKKDGKTVKEAHERLKSGSIDVLHCYYAHGEENINFQRRSYWML
EEDYMHIVLVHYLEVKAGKSSSRTRGHDNMLQGAYVDSPLSHLPSQSTDGESSLSGRASE
YEAESADIYSGGAGYHSISRMQQHENGGGSIIDASVVSSYSPASSVGNHQGLRATSPNTG
FYSHYQDNSPVIHNESTLGITFNGPSTQFDLSSWNEMTKLNKGIHQLPPYQSHVPSEQPP
FTEGPGIESFSFDEVYSNGLDIKDGGHADTDREALWQLPSAIDGRTTEFQLPSATDSTFA
TVDSFEQNNKLLEEAINFPVLKTQSSNLSDILKDSFKKSDSFTRWMSKELAEVDDSQVKS
SSGLYWNSEDADNIIGASSRDQLDQFTLDPMVAQDQLFSITEYFPSWTYAGSKTRVLVTG
RFLTSDEVIKLKWSCMFGEVEVPADILADGTLRCYSPSHKPGRVPFYVTCSNRLACSEVR
EFEYRPSDSQYMDAPSPHGATNKIYLQARLDELLSLGQDEQDEFQAALSNPTKELIDLNK
KITSLMTNNDQWSELLKFADDNQLAPDDRQDQFVESGMKEKLHIWLLRKAGGGGKGPSVL
DDEGQGVLHLAAALGYDWAIRPTITAGVSINFRDVHGWTALHWAAFCGRERTVVALIALG
AAPGALTDPRPDFPSGRTPADLASFNGHKGISGFLAEFSLTSHLQTLNLKEAMGSNASEV
SGLPGIGDVTGRIASPSAGQGLQAGSMGDSLGAVRNAAQAAARIYQVFRVQSFQRKQAVQ
YEDDNGVISDERAMSLLSYKPSKPGQFDPMHAAATRIQNKFRGWKGRKEFLLIRQRIVKI
QAHVRGHQVRKHYRKIIWSVGIVEKVILRWRRRGAGLRGFRSTEGATDSSTRSSSVDVTP
VKPAEDDYNFLQEGRKQTEERLQRALARVKSMVQYPEARDQYQRILTVVTKMQESQPVEE
SMLEESTEMDEGFLMSEFKELWDDDMPPLPGYF
>TaCAMTA1b-B.2
MEQILKEAQTRWLRPTEICEILKNYRNFHIAPEPPNMPASGSLFLFDRKVLRLFRKDGHN
WRKKKNGTTVKEAHERLTSGSIDVLHCYYAHGEDNINFQRRSYWMLEEDYMHIVLVHYLE
VKRAPCSSSRSSSTTGIGNLSLSVTALRARAQEVDPRMKSKQPKSMLAQVMVKCSSPILP
SGNDGTSTECLQLPSAIDGTTTEFQLPSATDSTFATVDSFEQNNKLLGEAINFPVLKTQS
SNLSDMLKDSFKRSDSFARWMSKELAEVDDSQVKSSSGLYRNSEDADNIIGASRHDQLDW
FTLDPMVAQDQLFSITEFFPSWTYAGSKTRVLVTGSFLTSDKVIKLKWSCMFGEVEVPAD
ILAGGTLRCYSPPHKPGRVPFYVTCSNKLACSEVREFEYRPSDSQYMDAPSPHGATNKIY
LQARLDELLSLGQDEQDKFQAALSGPKKELIDLNKKITSLMTDNDPWSELLKFADDNQLA
PDDRQDQFVESSIKEKLHIWLLHKAGGGGKDPSVLDEEGQGVLHLAAALGYDWVIRPTIT
AGVSINFRDVHGWTALHWAAFCGRERTVVALIALGAAPGALTDPRPDFPSGRTPADLASF
NGHKGISGFLAEFSLTSHLQTLNLKEAMGSNASEISGLPGIGDVTGRIASPSAGQGLQAG
LMGDSLGAVRNAAQAAARIFQVFRVQSFQRKQAVQYEDDNGVISYERAMSLLSYKPSKPG
QFDPMHAAAARIQNKFRGWKGRKEFLLIRQRIVKIQAHVRGHQVSKHYRKIIWSIGIMEN
VILRWRQQGAGLRGFRSTEGATDSSTSSSSVDVIPVKPAEDDYNFLQDGRKQAEERLQRA
LARVKSMVRYPEARDQDQRILTVVTKMQESQLVEETLLEESTEMDEGFFDE

>TaCAMTA1b-D
MAEGRRYGIAPQLDMEQILKEAQTRWLRPTEICEILKNYRNFRIAPEPPNMPASGSLFLF
DRKVLRFFRKDGHNWRKKKDGKTVKEAHERLKSGSIDVLHCYYAHGEENINFQRRSYWML
EEDYMHIVLVHYLEVKAGKSSSRTRGHDNMLQGAYVDSPLSHLPSQSTDGESSLSGRASE
YEAESADIYSGGAGYHSISRMQQHENGGGSIIDASVVSSYSPASSVGNHQGLQATSPNTG
FYSHYQDNSPVIHNESTHGITFNGPSTQFDLSSWNEMTKMNKGIHQLPPYQSHVPSEQRP
FTEGPGIESFSFDEVYSNGLDIKDDGHADTDREALWQLPSANDGTTTEFLQLPSAIDGRT
TEFQLPSATDSTFATVDSFEQNNKLLEEAINFPVLKTQSSNLSDILKNSFKKSDSFTRWM
SKELAEVDDSQVKSSSALYWNSEDADNIIGASSRDQLDQFTLDPMVAQDQLFSITEYFPS
WTYAGSKTRVLVTGRFLTSDEVIKLKWSCMFGEVEVPADILADGTLRCYSPSHKPGRVPF
YVTCSNRLACSEVREFEYRPSDSQYMDAPSPHGATNKIYLQARLDELLSLGQDEQDEFQA
ALSNPTKELIDLNKKITSLMTNNDQWSELLKFADDNQLAPDDRQDQFVESGIKEKLHIWL
LHKAGGGGKGPSVLDDEGQGVLHLAAALGYDWAIRPTITAGVSINFRDVHGWTALHWAAF
CGRERTVVALIALGAAPGALTDPRPDFPSGRTPADLASFNGHKGISGFLAEFSLTSHLQT
LNLKEAMGSNASEISGLPGIGDVTGRIASPSAGQGLQAGSMGDSLGAVRNAAQAAARIYQ
VFRVQSFQRKQAVQYEDDNGVISDERAMSLLSYKPSKPGQFDPMHAAATRIQNKFRGWKG
RKEFLLIRQRIVKIQAHVRGHQVRKHYRKIIWSVGIVEKVILRWRRRGAGLRGFRSTEVA
TDSSTSSSSVDVIPVKPAEDDYNFLQEGRKQTEERLQRALARVKSMVQYPEARDQYQRIL
TVVTKMQESQPVEESMLEESTEMDEGFLMSEFKELWDDDVPLPGYF
>TaCAMTA4a-A
MIDCFNSCITKCYLHFGPGTKPTTTLSYLPVLPLVGFDINVLLREAKSRWLKPSEVYYIL
LNHEQLQITHEPLNKPPSGALFLYNRRVNRFFRKDGYAWRRKKDGRTVGEAHERLKVGNI
DALSCYYAHGEQNPYFQRRCFWMLEPAYDHIVLVQYREVAEGRYYSTLSNGSAGSLSTLS
YPNDIHGKHGSTSDFSEGNESHQSSVTEVSSYSANKEYNHDSGVLLSIPELQQSTVMGMP
ELDQSSLERSSEFCMVNNNDSTNTSGLNQALKSIAEQLSLGDDDYIYINQARSLDFTTNT
EAADVQGNQTSNSLGDDEANQIRPEGAHGVGRGISSSWENVLQSDLGLPASSTYQFGAHY
QQSSEYQPPGGLDGSNLQLQISAAKRFLLGSEDPIDSPSYNFIPRDEGINGINTLSAHDS
SLESCLNPDWQRTTPVTLQSSSYQSNSCGYEISEFFDNGQFEPSSEEDTRLALKQKQQFS
IREISPEWAFCYEITKVIITGDFLCDPSNICWAVMFGDTEVPVEIVQPGVLRCHTPLHSA
GKLTLCITTGNRKVCSEIKDFEFRAKSTASSFTDFAPSSMKSTEELSLLAKFARILLCDN
GSSAASGDDPQPGQSPKLKMNEDNWQRLINELDVGCENPLSRVDWIMEELLKSKLQQWLS
LRLQGDDGTCSLSKNEQGIIHLISALGYDWALSSVLSAGVGINLRDSNGWTALHWAAYYG
REKMVAALLAAGASAPAVTDPTAQDPVGKSAAFLASERGHVGLAGYLSEVALTSYLASLT
IEESGISEGLAAIKAERAVESISQRSAQLHGGTEDELSLKDSLAAVRNAAQAAARIQNAF
RAFSFRRRQHKDARLKDEYGMTQEDIDELAAASRLYYQHHVSNGQFCDKAAVSIQKKYRG
WKGRKNFLQMRRNVVKIQAHVRGHQVRKKYKTFVSTVSVLEKVILRWRRKGHGLRGFRAE
QSVMIEAEEGEEEDDDDFEDDEAVKIFRRQKVDESVKESVSRVLSMVDSPEARMQYRRML
EEFRQATAELGASDKATSSILDNDLLVEINKFTC
>TaCAMTA4a-B
MSQSFDINVLLREAKSRWLKPSEVYYILLNHEQLQITHEPPNKPPSGALFLYNRRVNRFF
RKDGYAWRRKKDGRTVGEAHERLKVGNIDALSCYYAHGEQNPYFQRRCFWMLEPAYDHIV

LVQYREVAEGRYYSTLSNGSAGSLSTLSYPNDIHGKHGSTSDFSEGNESHQSSVTEVSSY
SANKEENHDSGVLLSIPELQQSTVMGIPELDQSSLERSSEFCMVNNNDSTNTSGLNQALK
SIAEQLSLGDDDYIYINQARSLDFTTNTEAADVQGNQTSNSLGDDEANQIRPEGAHGVGR
GISSSWENVLQSDLGLPASSTYQFGAHYQQSSEYQPPGGLDSSNLQLQISAAKRFLLGSE
DPIDSPSYIPRDEGINGINTLSAHDSSLESCLNPDWHRTTPVTLQSSLYQSNSCGYEISE
FFDNGQFEPSSEEDTRLTVKQKQQFSIREISPEWAFCYEVTKVIITGDFLCDPSNICWAV
MFGDTEVPVEIVQPGVLRCHTPLHSAGKLTLCITTGNRKVCSEIKDFEFRAKSTASSFID
FAPSSMKSTEELSLLAKFARILLCDNGSSAASGDDPQPGQSPKLKMNEDNWQRLINELDV
GCENPLSRVDWIMEELLKSKLQQWLSLRLQGDDGTCSLSKNEQGIIHLISALGYDWALSS
VLSAGVGINLRDSNGWTALHWAAYYGREKMVAALLAAGASAPAVTDPTAQDPVGKSAAFL
ASEQGHVGLAGYLSEVALTSYLASLTIEESGISDGLAAIEAERAVESISQRSAQLHGGTE
DELSLKDSLAAVRNAAQAAARIQNAFRAFSFRRRQHKDARLKDEYGMTQEDIDELAAASR
LYYQHHVSNGQFCDKAAVSIQKKYRGWKGRKNFLHMRRNVVKIQAHVRGHQVRKKYKTFV
STVSVLEKVILRWRRKGHGLRGFRAEQSVMIEAEEGEEEDDDDFDDDEAVKIFRRQKVDE
SVKESVSRVLSMVDSPEARMQYRRMLEEFRQATVSSSWI
>TaCAMTA4a-D
MIDCFNSCITKCFDINVLLREAKSRWLKPSEVYYILLNHEQLQITHEPLNKPPSGALFLY
NRRVNRFFRKDGYAWRRKKDGRTVGEAHERLKVGNIDALSCYYAHGEQNPYFQRRCFWML
EPAYDHIVLVQYREVAEGRYYSTLSNGSAGSLSTLSYPNDIHGKHGSTSDFSEGNESHQS
SVTEVSSYSANKEYNHDSGVLLSIPELQQSTVMGMPELDQSSLERSSEFCMVNNNDSTNT
SGLNQALKSIAEQLSLGDDDYIYINQARSLDFTTNTEAADVQGNQTSNSLGDDEANQIRP
EGAHGVGRGISSSWENVLQSDLGLPASSTYQFGAHYQQSSEYQPPGGLDGSNLQLQISAA
KRFLLGSEDPIDSPSYNFIPRDEGINGINTLSAHDSSLESCLNPDWQRTTPVTLQSSSYQ
SNSCGYEISEFFDNGQFEPSSEEDTRLALKQKQQFSIREISPEWAFCYEITKVIITGDFL
CDPSNICWAVMFGDTEVPVEIVQPGVLRCHTPLHSAGKLTLCITTGNRKVCSEIKDFEFR
AKSTASSFTDFAPSSMKSTEELSLLAKFARILLCDNGSSAASGDDPQPGQSPKLKMNEDN
WQRLINELDVGCENPLSRVDWIMEELLKSKLQQWLSLRLQGDDGTCSLSKNEQGIIHLIS
ALGYDWALSSVLSAGVGINLRDSNGWTALHWAAYYGREKMVAALLAAGASAPAVTDPTAQ
DPVGKSAAFLASERGHVGLAGYLSEVALTSYLASLTIEESGISEGLAAIKAERAVESISQ
RSAQLHGGTEDELSLKDSLAAVRNAAQAAARIQNAFRAFSFRRRQHKDARLKDEYGMTQE
DIDELAAASRLYYQHHVSNGQFCDKAAVSIQKKYRGWKGRKNFLQMRRNVVKIQAHVRGH
QVRKKYKTFVSTVSVLEKVILRWRRKGHGLRGFRAEQSVMIEAEEGEEEDDDDFEDDEAV
KIFRRQKVDESVKESVSRVLSMVDSPEARMQYRRMLEEFRQATAELGASDKATSSILDND
LLVEINKFTC
>TaCAMTA4b-A
MQQQQRQGHDILNLQREVKTRWLKPREVLDILQNCELFGIQNRTPQRPPSGSWFLFNRRV
HRFFRNDGYVWQKKKNGKSGNEAHEYLKVDNVKALNCYYARAENNPRFMRRIYWMLEPAY
EHIVLVHYRDVLEGSISVSVLNGSPTSNQNGSASRADAHSSPGLTSEIVAPLLNSCSPGS
AEEVSSQIPTINNETNDISLFDWRRTLEMQLSLENKERHDVNTDEVLPNHDPIPAHGIQN
EELDACINLADVFDLGFSEDNHAEGSHPYPDPIDVLKYSETWLEDDQLKSILHSAPVTID
ENQWFHLHEVSPEWAFCSESVVIVGDFPCNLSNSSWVLFGDVKVPAVVVQQGVIRCYTPP

YLGAGKVRMCMLNENGKPCTEDREFEFVEKPTNTMINGNGKPCSEAREFEFQQRPTKSDN
GLLLLINYVQMLFDSHGCELFSKFRLPLPNARSGFPVNPSEIIGRTCEQLDHENAVNCIM
EVMLNNKFQDWLSSKFEQNSEGEYLLPKQYHGVIHTIAALGYDWALKPLLSNGVPINYRD
ANGWTALHWAARFGREQMVAVLVVAGAAVGALSDPTAEDPAAKTPASIASAYGFIGISAF
LSEAQLTSTLDSLESKENGKPVDHNGGVSTSNAVDRVSDKCAHVDGGTDDQLALKDSLGA
IRNAVQAAGRIQATFRVFSLKKKKQKALQNGDSSASPSMLERATLSIQKNFRRWKKRKEY
QKIRKNVIKIQARFRAHRERNKYKELLQSVGILEKIMLRWFRKGVGLRGINSRAMPIDQD
EEEDIVKVFRKERVETAVSEAVSRVSAIVGCPVARLDYRRMLEMHQQAKIGHGK
>TaCAMTA4b-B
MQQQQQGLDIQNLQREVKTRWLKPREVLDILRNCELFGIQNRTPQRPPSGSWFLFNRRVH
RFFRNDGYQWQKKRNGKSGNEAHEYLKVDNVKALNCYYARAENNPTFMRRIYWMLEPAYE
HIVLVHYRDVLEGSISVSALNGSPTSYQNGSASRADAHSSPGLTSEIIAPRLLSRSPGSA
EEVSSQILTINNESNDTSQFDWRRMLEMQLSLENKERHDVTGEILPNHDPNPIPGIQNEE
FDTGTNLADIFYELEEFSEDNRTEGSQPYRDPIDVIRNSAWLEEDQLNSFLHSPPVTVDE
NQWFHIYEVSPEWAFCSESAKVVIAGDFPSNILWVLFGDVKVPAEIVQQGVIRCYTPSYL
GAGKVRMCMLDENGKPCTEAREFGFVEKPTNTMIIGNGKPCSEAREFEFQQRPTKSDNEL
LLLLNYVQMLFDSHGCELFSKFRSPLPNVQSGFPVNPSEIIGRTCEQLDHENAVNCIMEV
MLNSKFEDWLSSKFEQNSEGVYLLPKQYHGVIHTIAALGYDWALKPLLNNGVPINYRDAN
GWTALHWAARFGRQQMVAVLVAAGAAVGALSDPTAEDPAAKTPASIASAYGFIGISAFLS
EAELTSTFHSLESQENGKPVDHNGGVSTSSAVDRVSDKCAHMDGGTDDQLALKDSLGAIR
NAVQAAGCIQATFRVFSLKKKKQKALQNGDSSASPSMLERAALSIQKNFRCWKKRKEYQK
VRKNVIKIQARFRAHRERNKYKELLQSVGILEKIMLRWFRKGVGLRGINSRAMPIDQDEE
EDIVKVFRKERVETAVSEAVSRVSAIVGCPVARLDYRRMLEMHQQAKIGHGK
>TaCAMTA4b-D
MQQQQGLDIQNLQREVKTRWLKPREVLDILRNCELFGIQNRTPQRPPSGSWFLFNRRVHR
FFRNDGYQWQKKRNGKSGNEAHEYLKVNNVKALNCYYARAENSPTFMRRIYWMLEPAYEH
IVLVHYRDVLEGSISVSALNGSPTSYQNGSASRADAHSSPGLTSEIIAPRLLSRSPGSAE
EVSSQILTINNESNDTSQFDWRRMLEMQLSLENKERHDVNTGEVLPNHDPNPMPGIQNEE
FDTGTNLADIFYELEEFSEDNRTEGSQPYRDPIDVIRNSAWLEEDQLNSFLHSAPVTVDE
NQWFHIHEVSPEWAFCSESAKVVIAGDFPSNILWVLFGDVKVPAEIVQQGVIRCYTPSYL
GAGKVRMCMLDENGKPCTQDREFGFVEKPTNTMIIGNGKPYSEAREFEFQQRPTKSDNEL
LLLLNYVQMLFDSHGCELFSKFRLPLPNVQSGFPVNPSEIIGRTCEQLDHENAVNCIMEV
MLNSKFEDWLSSKFEQNSEGEYLLPKQYHGVIHTIAALGYDWALKPLLNNGVPINYRDAN
GWTALHWAARFGRQQMVAVLFAAGAAVGALSDPTAEDPAAKTPASIASAYGFIGISAFLS
EAELTSTLHSLESQENGKPVDHNGGVSTSSAVDRVSDKCAHVDGGTDDQLALRDSLGAIR
NAVQAAGCIQATFRVFSLKKKKQKALQNGDSSASPSMLERAALSIQKNFRCWKKRKEYQK
VRKNVIKIQARFRAHRERNKYKELLQSVGILEKIMLRWFRKGVGLRGINSRAMPIDQDEE
EDIVKVFRKERVETAVSEAVSRVSAIVGCPVARLDYRRMLEWHQQAKIGHGK
>TaCAMTA5-A
MAGAAGRERDPLLRSEIHGFITYADLNFEKLKAEAASRWFRPNEIYAVLANHERFKVHAQ
PIDKPVSGTIVLYDRKVVRNFRKDGHNWKKKKDGKTVQEAHEKLKIGNEERVHVYYARGE

DNPNFFRRCYWLLDKEAERIVLVHYRQTSEENAIAHPSTEAEAEAPTMNVIQYYTSPISA
NSASVHTEISFSPPAPEEINSHGGSAISSDTGGSSLEEFWVHLLESSMTKDTACASVAFS
QQIKCGTKDSGNDTDSTNNVHVNHAGALEHQVDQSQYPLTSDLDSQSHQFATSLRKTPVD
GDIPNDVPARENSLGLWKYLDDDSPCLGDNIVSNGKIFNITDFSPEWACSTEHTKILVIG
DYYEQYKHLAGSNIYGIFGDNCVAANMVQTGVYRFMVGPHTAGRVDFYLTLDGKTPISEV
LNFEYRSVPGSSLHSELKPLEDEYTKSKLQMQMRLARLLFVTNKKKIAPKLLVEGSKVSN
LILASPEKEWMDLWKIAGDSEGTSVHATEDLLELVLRNRLQEWLLERVIGGHKSTGRDDL
GQGPIHLCSFLGYTWAIRLFSVSGFSLDFRDSSGWTALHWAAYHGREKMVAALLSAGANP
SLVTDPTAVSPGGCTPADLAARQGYVGLAAYLAEKGLTAHFESMSLSKGTKQSPSRTKLT
KVHSEKFENLTEQELCLKESLAAYRNAADAASNIQAALRDRTLKLQTKAILLANPEMQAT
AIVAAMRIQHAFRNYNRKKEMRAAARIQNHFRTWKVRRNFKNMRRQAIRIQAAYRGHQVR
RQYRKVIWSVGVVEKAILRWRKKRKGLRGIANGMPAEMTVDVEAASTAEEGFFQASRQQA
EDRFNRSVVRVQALFRCHRAQHEYRRMRIAHEEAKLEFSKGQQQAPACRR

>TaCAMTA5-B.1

RLDQENAIAHPSTEAEAEAPTMNVIQYYTSPPISANSASVHTEISFSPPAPEEINSHGGS
AISSDTGGSSLEEFWVHLLESSMKKDTACGASVAFSQQIKCGMKDSGNDTDSTNNVHANH
AGALEHQLDQSQYPLTSDLDSQSQQFATSLRRTPVDGDIPNDVPARENSLGLWKYLDDDS
PCLGDNIVSTEKIFNITDFSPEWACSTEHTKILVIGHYYEQYKHLAGSNIYGIFGDNCVA
ANMVQTGVYRFMVGPHTAGRVDFYLTLDGKTPISEVLNFEYRSVPGSSLHSELKPLEDEY
TKSKLQMQMRLARLLFVTNKKKIAPKLLVEGSKVSNLILASPEKEWMDLWKIAGDSEGKS
VHATEDLLELVLRNRLQEWLLERVIGGHKSTGRDDLGQGPIHLCSFLGYTWAIRLFSVSG
FSLDFRDSSGWTALHWAAYYGREKMVAALLSAGANPSLVTDPTAVSPGGCTPADLAARQG
YVGLAAYLAEKGLTAHFESMSLSKGTKQSPSRTKLTKVHSEKFENLTEQELCLKESLAAY
RNAADAASNIQAALRDRTLKLQTKAILLANPEMQATAIVAAMRIQHAFRNYNRKKEMRAA
ARIQNHFRTWKVRRNFTNMRRQAIRIQAAYRGHQVRRQYRKVIWSVGVVEKAILRWRKKR
KGLRGIANGMPVEMTVDIEAASTAEEGFFQASRQQAEDRFNRSVVRVQALFRCHRAQHEY
RRMRIAHEEAKLEFSKGQQQAPACRR

>TaCAMTA5-B.2

MAGAAGRERDPLLRSEIHGFITYADLNFEKLKAEAASRWFRPNEIYAVLANHERFKVHAQ
PIDKPVSGTIVLYDRKVVRNFRKDGHNWKKKKDGKTVQEAHEKLKIGNEERVHVYYARGE
DNPNFFRRCYWLLDKEAERIVLVHYRQTSEENAIAHPSTEAEAEAPTMNVIQYYTSPISA
NSASVHTEISFSPPAPEEINSHGGSAISSDTGGSSLEEFWVHLLESSMTKDTACGASVAF
SQQIKCGTKDSGNDTDSTNNVHVNHAGALEHQVDQSQYPLTSDLDSQSHQFATSLRKTPV
DGDIPNDVPARENSLGLWKYLDDDSPCLGDNIVSNGKIFNITDFSPEWACSTEHTKILVI
GDYYEQYKHLAGSNIYGIFGDNCVAANMVQTGVYRFMVGPHTAGRVDFYLTLDGKTPISE
VLNFEYRSVPGSSLHSELKPLEDEYTKSKLQMQMRLARLLFVTNKKKIAPKLLVEGSKVS
NLILASPEKEWMDLWKIAGDSEGTSVHATEDLLELVLRNRLQEWLLERVIGGHKSTGRDD
LGQGPIHLCSFLGYTWAIRLFSVSGFSLDFRDSSGWTALHWAAYHGREKMVAALLSAGAN
PSLVTDPTAVSPGGCTPADLAARQGYVGLAAYLAEKGLTAHFESMSLSKGTKQSPSRTKL
TKVHSEKFENLTEQELCLKESLAAYRNAADAASNIQAALRDRTLKLQTKAILLANPEMQA
TAIVAAMRIQHAFRNYNRKKEMRAAARIQNHFRTWKVRRNFKNMRRQAIRIQLEFSKGQQ

QAPACRR
>TaCAMTA5-D
MEGAAGRERDPLLRSEIHGFITYADLNFEKLKAEAASRWFRPNEIYAVLANHERFKVHAQ
PIDKPVSGTIVLYDRKVVRNFRKDGHNWKKKKDGKTVQEAHEKLKIGNEERVHVYYARGE
DNPNFFRRCYWLLDKEAERIVLVHYRQTSEENAIAHPSTEAEAEAPTMNVIQYYTSPISA
NSASVHTEISFSPLAPEEINSHGGSAISSDTGGSSLEEFWVHLLESSMKKDTACGASVAF
SQQIKCGTKNSGNDTDSTNNVHANHAGALEHQVDQSQYPLTSDLDSQSQQFATSLRKTPV
DGDIPNDVPARENSLGLWKYLDDDSPCLGDNIVSNGKIFNITDFSPEWACSTEHTKILVI
GDYYEQYKHLAGSNIYGIFGDNCVAANMVQTGVYRFMVGPHTAGRVDFYLTLDGKTPISE
VLNFEYRSVPGSSLHSELKPLEDEYTKSKLQMQMRLARLLFVTNKKKIAPKLLVEGSKVS
NLILASLEKEWMDLWKIAGDSEGTSVHATEDLLELVLRNRLQEWLLERVIGGHKSTGRDD
LGQGPIHLCSFLGYTWAIRLFSVSGFSLDFRDSSGWTALHWAAYHGREKMVAALLSAGAN
PSLVTDPTAVSPGGCTPADLAARQGYVGLAAYLAEKGLTAHFESMSLSKGTKQSPSRTKL
TEVHSEKFENLTEQELCLKESLAAYRNAADAASNIQAALRDRTLKLQTKAILLANPEMQA
TAIVAAMRIQHAFRNYNRKKEMRAAARIQNHFRTWKVRRNFKNMRRQAIRIQAAYRGHQV
RRQYRKVIWSVGVVEKAILRWRKKRKGLRGIANGMPVEMTVDVEAASTAEEGFFQASRQQ
AEDRFNRSVVRVQALFRCHRAQHEYRRMRIAHEEAKLEFSKGQQQAPACRRTILLILLAE
SGVCICC
>NtCAMTA1
QLLLFYFSGYDINDLVREAQIRWLKPAEVLFILRNHENHQLSNEAAQKPPSGSLFLFNKR
VLRFFRKDGHSWRKKKDGRTVGEAHERLKVGNAEALNCYYAHGEQNPTFQRRSYWMLDPA
YEHIVLVHYRDITEGRQNPAFMSESSPISSTFSPSPSSYSTQQTGSTLIAGESYEQYQNQ
SSPGEICSDAVINNNGMSDIIGRTKEVMSSPGLEMSQALRRLEEQLSLNDDSFKEIDPLY
ADAISDDSSLVEMQGNSNSLLLQHHSAESSESHHQHLTQDGHVWKDMLDHYGVSTAAESL
TKSLPKLDENGMLQISSERGAIEAYQSYKWPNFSEKEAQKAPIPAFKQLENFKYPAYSPG
VTAFGSNSDQCTTIFDQDQIGTSFEDEMSLTISQKQKFTFRDISPDWGYSSEATKVVIIG
SFLCNPSECMWTCMFGDSEVPVQIIQEGVICCQAPPHLPGKVTLCVTSGNRESCSEVKEF
EYRDKPDDCARNNRSDVEGAYKSTEELLLLVRFVQLLLLDLSAQKEDSSMLSNDFLEKCK
ANEDSWSQVIESLLFGTSTSTITIDWLLQELLKDKFQQWLSYKLQRKDNQMGCSLSKKEQ
GIIHMVSGLGFEWALHPILNAAVSVNFRDINGWTALHWAARFGREKMVASLIASGASAGA
VTDPSSRDPVGKTAASIASSCGHKGLAGYLSEVALTSHLSSLTLEESELSKGTADVEAEK
TISSISNTSATTNEDQRSLKDSLAAVRNAAQAAARIQSAFRAHSFRKRQQRESAVTATAS
GDEYGILSNNIHGLSAASKWAFRNTRDYNSAALAIQKKYRGWKGRKDFLAFRQKVVKIQA
HVRGYQVRKQYKVCWAVGILEKVVLRWRRRGVGLRGFRHDTESIDESEDEDILKVFRKQK
VDAALDEAVSRVLSMVESPGARQQYHRILEKYRQAKAELEGAESESASTAHGDMSNMEND
DIYQFSSY
>NtCAMTA2
MADSRRYGLNAQLDIDQILLEAQHRWLRPAEICEILKNYQKFRIAPEPPNRPPSGSLFLF
DRKVLRYFRKDGHSWRKKKDGKTVKEAHERLKAGSIDVLHCYYAHGEENENFQRRSYWML
EEEMSHIVLVHYREVKGNRTNFSRTREPQEAAPRFQETDEDVHSSEVDSSASTKFYPNDY
QVNSQVTDTTSLSSVQASEYEDAESAYNQHPTSGFHSFLDAQPSMTQKAGEGLAVPYHPI

PFSTDDHQVQFAGSSDMDFFSIAPGNKSGNTANTYIPSRNLDFPSWETTSVNNPAAYQSY
HFQPSSQSGANNMTHEQGNTKTGQVFLNDFKRQERQNRIDGLGDWQTSEGDAAFISKWSM
DQKLHPDLASDHTIRSSAAYNVELHNSLEASHILPSHQDKHPMQNELPSQLSDPNVGGSL
NADLDHNLSIGVRTDHSSLKQPLLDGVLREGLKKLDSFDRWMSKELEDVSEPHMQSNSSS
YWDNVGDDDGVDNSTIASQVQLDTYMLSPSLSQDQFFSIIDFSPSWAFAGSEIKVLITGK
FLKSQPEVEKCSWACMFGELEVPAEVIADGVLRCHTPIQKAGRVPFYITCCNRLACSEVR
EFEFRVTEGQDADVANANSCSSSESLLHMRFGKLLSLESTVSLSSPPRSEDDVSHVCSKI
NSLLNEDDNEWEEMLNLTYENNFMAEKVKDQLLQKLLKEKLRVWLLQKVAEGGKGPNVLD
EGGQGVLHFAAALGYDWAIPPTIAAGVSVNFRDVNGWTALHWAASYGRERTVGFLISLGA
APGALTDPTPKHPSGRTPADLASSNGHKGIAGYLAESSLSFHLSSLELKEMKQGENVQPF
GEAVQTVSERSATPAWDGDWPHGVSLKDSLAAVRNATQAAARIHQVFRVQSFQRKQLKEH
GGSEFGLSDEHALSLLALKTNKAGQHDEPVHTAAVRIQNKFRSWKGRRDYLLIRQRIIKI
QAHVRGHQVRNKYKNIIWSVGILEKVILRWRRKGSGLRGFKPEATLTEGSDTQDRPVQED
DYDFLKEGRKQTEQRLQKALARVKSMVQYPEARDQYRRLLNVVSDMKDTTTTSDGAPSNS
VEAADFGDDLIDLDDLLDDDTFMSTAP

>NtCAMTA3

MADSRRYGLNAQLDIDQILLEAQHRWLRPAEICEILKNYQKFRIAPEPPNRPPSGSLFLF
DRKVLRYFRKDGHSWRKKKDGKTVKEAHERLKAGSIDVLHCYYAHGEENENFQRRSYWML
EEEMSHIVLVHYREVKGNRTNFSRTREPQEAAPRFQETDEDVHSSEVDSSASTKFYPNDY
QVNSQVTDTTSLSSVQASEYEDAESAYNQHPTSGFHSFLDAQPSMTQKAGEGLAVPYHPI
PFSNDHQVQFAGSSDMDFFSIAPGNKSGNTANTYIPSRNLDFPSWETTSVNNPAAYQSYH
FQPSSQSGANNMTHEQGNTKTGQVFLNDFKRQERQNRIDGLGDWQTSEGDAAFISKWSMD
QKLHPDLASDHTIRSSAAYNVELHNSLEASHILPSHQDKHPMQNELPSQLSDPNVGGSLN
ADLDHNLSIGVRTDHSSLKQPLLDGVLREGLKKLDSFDRWMSKELEDVSEPHMQSNSSSY
WDNVGDDDGVDNSTIASQVQLDTYMLSPSLSQDQFFSIIDFSPSWAFAGSEIKVLITGKF
LKSQPEVEKCSWACMFGELEVPAEVIADGVLRCHTPIQKAGRVPFYITCCNRLACSEVRE
FEFRVTEGQDADVANANSCSSSESLLHMRFGKLLSLESTVSLSSPPRSEDDVSHVCSKIN
SLLNEDDNEWEEMLNLTYENNFMAEKVKDQLLQKLLKEKLRVWLLQKVAEGGKGPNVLDE
GGQGVLHFAAALGYDWAIPPTIAAGVSVNFRDVNGWTALHWAASYGRERTVGFLISLGAA
PGALTDPTPKHPSGRTPADLASSNGHKGIAGYLAESSLSFHLSSLELKEMKQGENVQPFG
EAVQTVSERSATPAWDGDWPHGVSLKDSLAAVRNATQAAARIHQVFRVQSFQRKQLKEHG
GSEFGLSDEHALSLLALKTNKAGQHDEPVHTAAVRIQNKFRSWKGRRDYLLIRQRIIKIQ
AHVRGHQVRNKYKNIIWSVGILEKVILRWRRKGSGLRGFKPEATLTEGSDTQDRPVQEDD
YDFLKEGRKQTEQRLQKALARVKSMVQYPEARDQYRRLLNVVSDMKDTTTTSDGAPSNSV
EAADFGDDLIDLDDLLDDDTFMSTAP

>NtCAMTA4

MADSRRYGLNAQLDIDQILLEAQHRWLRPAEICEILKNYQKFRIAPEPPNRPPSGSLFLF
DRKVLRYFRKDGHSWRKKKDGKTVKEAHERLKAGSIDVLHCYYAHGEENENFQRRSYWML
EEEMSHIVLVHYREVKGNRTNFSRTREPQEAAPRFQETDEDVHSSEVDSSASTKFYPNDY
QVNSQVTDTTSLSSVQASEYEDAESAYNQHPTSGFHSFLDAQPSMTQKAGEGLAVPYHPI
PFSRNKSGNTANTYIPSRNLDFPSWETTSVNNPAAYQSYHFQPSSQSGANNMTHEQGNTK

TGQVFLNDFKRQERQNRIDGLGDWQTSEGDAAFISKWSMDQKLHPDLASDHTIRSSAAYN
VELHNSLEASHILPSHQDKHPMQNELPSQLSDPNVGGSLNADLDHNLSIGVRTDHSSLKQ
PLLDGVLREGLKKLDSFDRWMSKELEDVSEPHMQSNSSSYWDNVGDDDGVDNSTIASQVQ
LDTYMLSPSLSQDQFFSIIDFSPSWAFAGSEIKVLITGKFLKSQPEVEKCSWACMFGELE
VPAEVIADGVLRCHTPIQKAGRVPFYITCCNRLACSEVREFEFRVTEGQDADVANANSCS
SSESLLHMRFGKLLSLESTVSLSSPPRSEDDVSHVCSKINSLLNEDDNEWEEMLNLTYEN
NFMAEKVKDQLLQKLLKEKLRVWLLQKVAEGGKGPNVLDEGGQGVLHFAAALGYDWAIPP
TIAAGVSVNFRDVNGWTALHWAASYGRERTVGFLISLGAAPGALTDPTPKHPSGRTPADL
ASSNGHKGIAGYLAESSLSFHLSSLELKEMKQGENVQPFGEAVQTVSERSATPAWDGDWP
HGVSLKDSLAAVRNATQAAARIHQVFRVQSFQRKQLKEHGGSEFGLSDEHALSLLALKTN
KAGQHDEPVHTAAVRIQNKFRSWKGRRDYLLIRQRIIKIQAHVRGHQVRNKYKNIIWSVG
ILEKVILRWRRKGSGLRGFKPEATLTEGSDTQDRPVQEDDYDFLKEGRKQTEQRLQKALA
RVKSMVQYPEARDQYRRLLNVVSDMKDTTTTSDGAPSNSVEAADFGDDLIDLDDLLDDDT
FMSTAP
>NtCAMTA5
MADCGSDPSGFRLDITQILSEVQHRWLRPAEICEILRNYKKFHITPEAPHRPVSGSVFLF
DRKVLRYFRKDGHNWRKKKDGKTVKEAHEKLKVGSIDVLHCYYAHGEEDDNFQRRSYWML
EQDLMHIVFVHYLEVKGNKANMGCVRSIKSAHSNYLNDCSLSDSFPRGHKKLASANADST
SVASTLTSAHEEAESEDSHQACSRFQSYPERASGMDRNLVENRDTIYSSYGSPQSSVEYT
SLPGIDVGEKCGLGNFASGPQRTIDLGSQEPVSQHCSNGEIVCQDDFKNNLSVKGNWQII
YLSSPCPCFPSFYVPXDLVNSFHNKNLSSDLYTGRGQSYLYPDEQEEQLTQLNIQYLNSL
VEVQGDFNQENSMDMLGLGDYYTIKQPHLNSVKMEEGLKKVDSFSRWVVKELEDVEELHM
QPTNRISWNVIDTLDDGSCLPTQLHVDSDSLNPSLSQEQVFSIIDFSPNWAYSNLETKVL
ITGRFLKSEGELIECKWSCMFGEIEVPAEVLADGVLRCHAPPHKPGVLPFYVTCSNRLAC
SEVREFEYRLGAYQEFGAANVSATEMHLLERIESLLSLEPLSSCHSSDSMEAAKEKQSTV
NRIICMMEEENQQMIERASDHDTSQCGVKEDLFLERKLKQNFYAWLVRQVTDDGRGRTAI
DDEGQGVLHLAAALGYDWALKPILASGVSVDFRDMNGWTALHWAAFYGREKTVVGLVSLG
ASPGALTDPSAEFPLGRTPADLASANGHKGISGFLAESSLTTHLSKLTVTDAKEELASEV
SGAKVGETVTERVAVTTTGDDMPDVLSLKDSLAAIRNATQAAARIHQIFRVQSFQRKQII
ECSDNELSSDENALSIVASRACKLGQNNGIAHAAATQIQKKFRGWNKRKEFLLIRQKIVK
IQAHVRGHQVRKKYKPIIWSVGILEKVILRWRRKRSGLRGFRSEVVMNKPIIQDDSLPED
DYDFLKEGRKHTEVRMQKALARVKSMTQYPEGRAQYRRLLTAAEGLREVKPDGPTCILES
PEDTSYPEEELFDVENLLDDDTFMSIAFE
>NtCAMTA6
MKFEVFIFAPFLLVTDTGRYLYNKSLDVGQILQEAQHRWFRPDEICEILRNHQKFGLTPQ
PPLRPPGGSLYLFDRKVLPYFCEDGHQWRKEEDGKAVKEIHEKLKAGSNDVLHCYYAHGE
DNENFQRRSYWMLEEPMEHIILVHYREVKEGYIVGASCLQPVHPGLLLENPQSSSAPCFE
SDLIVQESHTSTPSSVDWKEYVLSSELHSGHAKRNEADPLLVPAGLVESSRDSFQLNSRF
SSLLSGEVLEENTHIAPPQIQNISISQTVVSPDTAVQISSLEGGVNSDEAGSLKKFDSYV
RWMDREKSRDCDESLMATKSGNYWNTLDTDNEGKSCNLSIQMEYERKRTDTQLPLAPFGH
SSFQYPFLSKDIITEILIRLPVKLIVKFRCVSKSWRALITSPKFVKDHLRLALKNKAYHK

VMWCHRPQIKIEECYLSSLFDNSTTFSDVSALHPRLVSDGLYTRVPDSIVVLGSVHGLIC
LIGSVDGCIFQGYDDIDTERLVDIEDFKEDLFLWNPSTRRYKKLPDHKPDYWFDTRVYGL
GYDMLHDDYKFLGTFRGYQRLHCCKVQLYSLKSDSWKSLDDLPSALSGGNLQGVSGTFVE
GSLHWLSYTTDGPDKRWNIISFNLADEKWGTLEKPCYEEGELVLWLGVIGSDLSLFVDCE
GTHIDVWVMQKYAVQGSWNKKFIIKYPDGVGLTGFGPLFMLPYFFSITDEILMLFNSTAN
IYNTRDEVWRPNPYVINWLDHPTAFIYVESLVSPFSAKGTEATRKNLLDVAADPTLPHTI
SVKCPQSDHKK
>NtCAMTA7
MADSRRYGLNAQLDIDQILLEAQHRWLRPAEICEILKNYQKFRIAPEPPNRPPSGSLFLF
DRKVLRYFRKDGHSWRKKKDGKTVKEAHERLKAGSIDVLHCYYAHGEENENFQRRSYWML
EEEMSHIVLVHYREVKGNRTNFSRTREPQEATPRFQETDEDVHSSEVDSSASTKFYPNGY
QVNSQVTDATSLSSAQASEYEDAESAYNQHPTSGFHSFLDAQPSMMQKAGESLPVPYHPI
PFSTDDHQVQFAGSSDMDFFSSAPGNKSRNTANTYIPSRNLDFPSWETISVNNPAAYQSY
HFQPSSQSGANNMTHEQGSTTMGQVFLNDFKKQGQNRIDSLGDWQTSEGDAAFISKWSMD
QKLNPNLASDHTIRSSAAYNVELHNSLEASHILPSHQDKHPMQNELPSQLSDANVGGSLN
AELDHNLSIGVRTDHSSLKQPLLDGVLREGLKKLDSFDRWMSKELEDVSEPHMQSNSSSY
WDNVGDDDGVDNSTIASQVQLDTYMLSPSLSQDQFFSIIDFSPSWAFAGSEIKVLITGKF
LKSQPEVEKWACMFGELEVPAEVIADGVLRCHTPNQKVGRVPFYITCSNRLACSEVREFE
FRVSESQDVDVANSCSSSESLLHMRFGKLLSLESTVSLSSPPRSEDDVSNVCSKINSLLK
EDDNEWEEMLNLTYENNFMAEKVKDQLLQKLLKEKLRVWLLQKVAEGGKGPNVLDEGGQG
VLHFAAALGYDWAIPPTIAAGVSVNFRDVNGWTALHWAASYGRERTVGFLIISLGAAPGA
LTDPTPKHPSGRTPADLASSNGHKGIAGYLAESSLSSHLSSLELKEMKQGETVQPFGEAV
QTVSERSATPAWDGDWPHGVSLKDSLAAVRNATQAAARIHQVFRVQSFQRKQLKEHGGSE
FGLSDEHALSLLALKTNKAGQHDEPVHTAAVRIQNKFRSWKGRRDYLLIRQRIIKIQAHV
RGHQVRNKYKNIIWSVGILEKVILRWRRKGSGLRGFKPEATLTEGSNMQDRPVQEDDYDF
LKEGRKQTEQRLQKALARVKSMVQYPEARDQYRRLLNVVSDMKDTTTTSDGAPSNSGEAA
DFGDDLIDLDDLLDDDTFMSTAP
>NtCAMTA8
MADSRRYGLNAQLDIDQILLEAQHRWLRPAEICEILKNYQKFRIAPEPPNRPPSGSLFLF
DRKVLRYFRKDGHSWRKKKDGKTVKEAHERLKAGSIDVLHCYYAHGEENENFQRRSYWML
EEEMSHIVLVHYREVKGNRTNFSRTREPQEATPRFQETDEDVHSSEVDSSASTKFYPNGY
QVNSQVTDATSLSSAQASEYEDAESAYNQHPTSGFHSFLDAQPSMMQKAGESLPVPYHPI
PFSNDHQVQFAGSSDMDFFSSAPGNKSRNTANTYIPSRNLDFPSWETISVNNPAAYQSYH
FQPSSQSGANNMTHEQGSTTMGQVFLNDFKKQGQNRIDSLGDWQTSEGDAAFISKWSMDQ
KLNPNLASDHTIRSSAAYNVELHNSLEASHILPSHQDKHPMQNELPSQLSDANVGGSLNA
ELDHNLSIGVRTDHSSLKQPLLDGVLREGLKKLDSFDRWMSKELEDVSEPHMQSNSSSYW
DNVGDDDGVDNSTIASQVQLDTYMLSPSLSQDQFFSIIDFSPSWAFAGSEIKVLITGKFL
KSQPEVEKWACMFGELEVPAEVIADGVLRCHTPNQKVGRVPFYITCSNRLACSEVREFEF
RVSESQDVDVANSCSSSESLLHMRFGKLLSLESTVSLSSPPRSEDDVSNVCSKINSLLKE
DDNEWEEMLNLTYENNFMAEKVKDQLLQKLLKEKLRVWLLQKVAEGGKGPNVLDEGGQGV
LHFAAALGYDWAIPPTIAAGVSVNFRDVNGWTALHWAASYGRERTVGFLIISLGAAPGAL

TDPTPKHPSGRTPADLASSNGHKGIAGYLAESSLSSHLSSLELKEMKQGETVQPFGEAVQ
TVSERSATPAWDGDWPHGVSLKDSLAAVRNATQAAARIHQVFRVQSFQRKQLKEHGGSEF
GLSDEHALSLLALKTNKAGQHDEPVHTAAVRIQNKFRSWKGRRDYLLIRQRIIKIQAHVR
GHQVRNKYKNIIWSVGILEKVILRWRRKGSGLRGFKPEATLTEGSNMQDRPVQEDDYDFL
KEGRKQTEQRLQKALARVKSMVQYPEARDQYRRLLNVVSDMKDTTTTSDGAPSNSGEAAD
FGDDLIDLDDLLDDDTFMSTAP
>NtCAMTA9
MADCGSDPPGFRLDITQILSEVQHRWLRPAEICEILRNYRKFHITPEAPHRPVSGSVFLF
DRKVLRYFRKDGHNWRKKKDGKTVKEAHEKLKVGSIDVLHCYYAHGEEDDNFQRRSYWML
EQDLMHIVFVHYLEVKGNKANVGCVRSIKSAHSNYLNDCSLSDSFPRSLKKLASVNADST
SVASTLTSAHEEAESEDSHQACSRFQSYPERASGMDRHLVENRDAIYSSYGSPQSSVEYT
SLSSIDGGGKCGRGNFASGPQRTIDLGSQEPVSQHCSNGEMVCQDDFKNNLSVQRNWQYS
FGDSASQFHGQIVNQDLIADSSYDLVNSFHNKNLSSDLYTGRGQSYLYPDEQEEQLTQLN
IQYLNSLVEVQGDFNQENSMDMLGLGDYSTIKHPHLNSVKMEEGLKKVDSFSRWVVKELE
DVEELHMQRTNRISWNVIDTEDDGSCLPTQLHVDSDSLNPSLSQEQVFSIIDFSPNWAYS
NLETKVLITGRFLKSEGELIECKWSCMFGEVEVPAEVLADGVLRCHAPPHKPGVLPFYVT
CSNRLACSEVREFEYRLGAYQEIGAANVSATEMHLLERIESLMSLGPVSSCHSSDSMEAA
KEKHSTVNKIICMMEEENQQMIERASDYDTSQCGVKEDLFLERKLKQNFYAWLVRQVTDD
GRGRTAIDDEGQGVLHLAAALGYDWALKPILASGVSVDFRDMNGWTALHWAAFYGREKTV
VGLVSLGASPGALTDPSAEFPLGRTPADLASANGHKGISGFLAESSLTTHLSKLTVTDAT
EELASEVSGAKVGETVTERVAVTTTGDDVPDVLSLKDSLAAIRNATQAAARIHQIFRVQS
FQRKQIIERSDNELSSDENALSIVASRACKLGQNNGIAHAAATQIQKKFRGWNKRKEFLL
IRQKIVKIQAHVRGHQVRKKYKPIIWSVGILEKVILRWRRKRSGLRGFRSEVVINKPSIQ
DDSLPEDDYDFLKEGRKQTEVRMQKALARVKSMTQYPEGRAQYRRLLTAAEGLREVKQDG
STCIQESSEDTSYPEEELFDVENLLDDDTFMSIAFE
>NtCAMTA10
MADCGSDPPGFRLDITQILSEVQHRWLRPAEICEILRNYRKFHITPEAPHRPVSGSVFLF
DRKVLRYFRKDGHNWRKKKDGKTVKEAHEKLKVGSIDVLHCYYAHGEEDDNFQRRSYWML
EQDLMHIVFVHYLEVKGNKANVGCVRSIKSAHSNYLNDCSLSDSFPRSLKKLASVNADST
SVASTLTSAHEEAESEDSHQACSRFQSYPERASGMDRHLVENRDAIYSSYGSPQSSVEYT
SLSSIDGGGKCGRGNFASGPQRTIDLGSQEPVSQHCSNGEMVCQDDFKNNLSVQRNWQYS
FGDSASQFHGQIVNQDLIADSSYDLVNSFHNKNLSSDLYTGRGQSYLYPDEQEEQLTQLN
IQYLNSLVEVQGDFNQENSMDMLGLGDYSTIKHPHLNSVKMEEGLKKVDSFSRWVVKELE
DVEELHMQRTNRISWNVIDTEDDGSCLPTQLHVDSDSLNPSLSQEQVFSIIDFSPNWAYS
NLETKVLITGRFLKSEGELIECKWSCMFGEVEVPAEVLADGVLRCHAPPHKPGVLPFYVT
CSNRLACSEVREFEYRLGAYQEIGAANVSATEMHLLERIESLMSLGPVSSCHSSDSMEAA
KEKHSTVNKIICMMEEENQQMIERASDYDTSQCGVKEDLFLERKLKQNFYAWLVRQVTDD
GRGRTAIDDEGQGVLHLAAALGYDWALKPILASGVSVDFRDMNGWTALHWAAFYGREKTV
VGLVSLGASPGALTDPSAEFPLGRTPADLASANGHKGISGFLAESSLTTHLSKLTVTDAT
EELASEVSGAKVGETVTERVAVTTTGDDVPDVLSLKDSLAAIRNATQAAARIHQIFRVQS
FQRKQIIERSDNELSSDENALSIVASRACKLGQNNGIAHAAATQIQKKFRGWNKRKEFLL

IRQKIVKIQAHVRGHQVRKKYKPIIWSVGILEKVILRWRRKRSGLRGFRSEVVINKPSIQ
DDSLPEDDYDFLKEGRKQTEVRMQKALARVKSMTQYPEGRAQYRRLLTAAEGLREVKDGS
TCIQESSEDTSYPEEELFDVENLLDDDTFMSIAFE
>NtCAMTA11
MAESGYDINDLVREAQIRWLKPAEVLFILRNHEYHQLSNEPAQKPPSGSLFLFNKRVLRF
FRKDGHSWRKKKDGRTVGEAHERLKVGNAEALNCYYAHGEQNPNFQRRSYWMLDPVYEHI
VLVHYRDITEGRQNPAFMSESSPISSTFSPSPSSYSTQQTGSAVIAGESYEQYQNQFSPG
EICSDAVINNNRTSDITGRTNEVMSSPGLEMSQALRRLEEQLSLNDDSFKEIDPLYADAI
SDDSSLVEMQGSSNSLLLQHHSAESSESHHQHLTQDGHIWKDMLDHYGVSTADESLNKSL
PKLDENGMLQISSERGAIEAYQSYKWPNFSEKEAQKAPIPAFKQLENFKYPAYSPGVTAF
GSNSDQCTTIFDQDQIGTSLEDEMSLTISQKQKFTIRDISPDWGYSSEATKVVIIGSFLC
NPSECMWTCMFGDTEVPIQIIQEGVICCQAPPHLPGKVTLCVTSGNRESCSEVKEFEYRV
KPDDCARNNRSDIEGAYKSTEELLLLVRFVQMLLLDLSVHKEDSSELSNDFLEKSKANED
SWSQVIESLLFGTSTSTITIDWLLQELLKDKFQQWLSYKLQRKDNQMGCSLSKKEQGIIH
MVSGLGFEWALHPILNAGVSVDFRDINGWTALHWAARFGREQMVASLIASGASAGAVTDP
SPRDPVGKTAASIASSCGHKGLAGYLSEVALTSHLSSLTLEESELSKGTADVEAEKTISS
ISNTSATTNEDQRSLKDSLAAVRNAAQAAARIQSAFRAHSFRKRQQRESAIATTASGDEY
GILSNDILGLSAASKWAFRNTRDYNSAALAIQKKYRGWKGRKDFLAFRQKVVKIQAHVRG
YQVRKQYKVCWAVGILEKVVLRWRRRGVGLRGFRH
>NtCAMTA12
MTELYNKQPTIAPVSSPNTKTVKYSRFQVGSGNRILNERGRERAILHHREILLLRISMAE
SGYNINNLVREGRFRWLRPAEVLFILQNHEDQQLANQPPQKPASGSMFLFNKRVLRYFRK
DGHSWRKKKDGRTVGEAHERLKVGNAETLNCYYAHGEKNPNFQRRSYWMLDPAYEHIVLV
HYRDITEGMQIAAFMSQSSPISSTFSLSPSLYSTQHPGFTVVGSESYQQYQNESSPGSGE
ICSGAGINSNGMNISDITGRTEGVSSSPQVEISQALRKLEEQLSLNETDPLYSEIENSDD
VENFGHDNSSLVQIQHKSNNLLLQPYSGESSESQHQLLNLDGDIWKEMLDHCRSFPAAES
QDKCFEKLDENGTLQTLSGMGPIEVTESDRWLKFGGKEALKSSLTNFKQVEDFKYPACAR
INTYGSYSDQYTTIFDQDLIGTSFEDDMSLTIAQKQKFTFHDISPDWGYSSEATKVMIVG
SFLCNPSEYTWTCMFGDIEVPVQIIKEGAIRCQAPPHLPAEVGGACKSSEELLHLVRFVQ
MLLSDSSVQKGDGSGSSNDILENSKASEDSWSQVIESLLFGTSTSMVTVDWLLQELLKDK
LQQWLSSKLQVQNNQMGYSFSRKEQGIIHMVAVLGFEWALQPILDAGVSVNFRDINGWTA
LHWAARFGREKMVASLVASGAFAGAVTDPSSQDPFGKTAASIASSCGHKGVAGYLSEVAL
TSHLSSLTLEESELSKGAADVEAERTISSISTTNAATHEDQLSLKDTLAAVRNAAQAAAR
IQSAFRAHSFRKRRQREAARAATTSGDEYCVLSNDVLGLSAASKLAFRNMRDYNSAALAI
QKKYRGWKCRKGFLAFRQKVVKIQVLYLFESRIDISTSFVTTYKIKDDRSKKKRCHQFRC
Y
>NtCAMTA13
MESSRAGQLAGSDIHGFHTLQDLDIPSIMEEAKMRWLRPNEIHAILCNYKYFNIFVKPVN
LPMSGTIVLFDRKMLRNFRKDGHNWKKKKDGKTVKEAHEHLKVGNEERIHVYYAHGEDHP
TFVRRCYWLLDKSLEHIVLVHYRETQEAQGSPATSVAKGSPATPVNSNSSSDPSDPSGWV
LSEKCNSVDERTYGSSQHAHLEPNRDVTAKNHEQRLLEINTLEWDELLAPDNPNKLIATQ

EAGGRASVGQQNQIEVNGYSLNDGSLSVSRVPVASLESFVCQVAGSDTVNFNPSNDMPFH
SGDGQMTSNFRKNEPGVTTVGAGDSFDSLNKDGLQTQDSFGRWINYFISDSPGSADEMMT
PESSVTIDQSYVMQQIFNITEISPTWALSSEETKILVIGHFPGAQSQLAKSNLFCVCADV
CFPAEFVQSGVYRCVISPQPPGLVSLYLSFDGNTPISQVMTYEFRAPSACKWTAPLEEQS
SWDEFRVQMRLAHLLFSTSKSLSIFSSKVHQDSLKEAKRFVRKCSHITDNWAYLIKSIED
RKLPVPHAKDCLFELSLQTKFHEWLLERVIGGCKTSEWDEQGQGVIHLCAILGYTWAVYP
FSWSGLSLDYRDKYGWTALHWAAHYGREKMVATLLSAGAKPNLVTDPTSENPGGSTAADL
ASKNGFEGLGAYLAEKALVAHFKDMTLAGNVSGSLQTTTEHINPGNFTEEELYLKDTLAA
YRTAADAAARIQAAFREHSFKVQTKAVESSNPEMEARNIVAAMKIQHAFRNYESRKKLAA
AARIQYRFRSWKMRKDFLNMRRHAIKIQAVFRGFQVRKQYRKIVWSVGVLEKAVLRWRLK
RKGFRGLQVQSSQAVDIKPDGDVEEDFFRASRKQAEERVERSVVRVQAMFRSKRAQEEYR
RMKLEHDNATLEYERASLLNPDIQIG

>NtCAMTA14

MQVGNEERIHVYYAHGEDHPTFVRRCYWLLDKSLEHIVLVHYRETQEAQGSPATSVAKGS
PATPVNSNSSSDPSDPSGWVLSEKCNSVDERTYGSSQHAHLEPNRDVTAKNHEQRLLEIN
TLEWDELLAPDNPNKLIATQEAGGRASVGQQNQIEVNGYSLNDGSLSVSRVPVASLESFV
CQVAGSDTVNFNPSNDMPFHSGDGQMTSNFRKNEPGVTTVGAGDSFDSLNKDGLQTQDSF
GRWINYFISDSPGSADEMMTPESSVTIDQSYVMQQIFNITEISPTWALSSEETKILVIGH
FPGAQSQLAKSNLFCVCADVCFPAEFVQSGVYRCVISPQPPGLVSLYLSFDGNTPISQVM
TYEFRAPSACKWTAPLEEQSSWDEFRVQMRLAHLLFSTSKSLSIFSSKVHQDSLKEAKRF
VRKCSHITDNWAYLIKSIEDRKLPVPHAKDCLFELSLQTKFHEWLLERVIGGCKTSEWDE
QGQGVIHLCAILGYTWAVYPFSWSGLSLDYRDKYGWTALHWAAHYGREKMVATLLSAGAK
PNLVTDPTSENPGGSTAADLASKNGFEGLGAYLAEKALVAHFKDMTLAGNVSGSLQTTTE
HINPGNFTEEELYLKDTLAAYRTAADAAARIQAAFREHSFKVQTKAVESSNPEMEARNIV
AAMKIQHAFRNYESRKKLAAAARIQYRFRSWKMRKDFLNMRRHAIKIQAVFRGFQVRKQY
RKIVWSVGVLEKAVLRWRLKRKGFRGLQVQSSQAVDIKPDGDVEEDFFRASRKQAEERVE
RSVVRVQAMFRSKRAQEEYRRMKLEHDNATLEYERASLLNPDIQIG

>NtCAMTA15

MESSRAGQLAGSDIHGFRTLQDLDIPSIMEEAKMRWLRPNEIHAILCNYKYFNIFVKPVN
LPTSGTIVLFDRKMLRNFRKDGHNWKKKKDGKTVKEAHEHLKVGNEERIHVYYAHGEDHP
TFVRRCYWLLDKSLEHIVLVHYRETQETQGSPVTSVAKGSPATPVNSNSSSDPSDPSGWV
LSEECNSVDERAYGSSQHAHLEPNRDMTAKNHEQRLLEINTLEWDELLAPENPNKLNATQ
EAGGRASAGQQNQFEVNGYSLNDGSLSVSRVPVASLESFVCQVAGSDTVNFNPSNDTSFR
SGDGQMTSNFQKNESGVTTVGAGDSFDSLNKDGLQTQDSFGRWINYFISDSPGSADEMMT
PESSVTIDQSYVMQQIFNITEISPTWALSSEETKILVIGHFPGGQSQLAKSNLFCVCADV
CFPAEFVQSGVYRCVISPQPPGLVNLYLSFDGNTPISQVMTYEFRAPSARKWTAPLEEQS
SWDEFRVQMRLAHLLFSTSKSLSIFSSKVHQDSLKEAKRFVRKCSHITDNWAYLIKSIED
RKLPVPHAKDCLFELSLQTKFHEWLLERVIGGCKTSEWDEQGQGVIHLCAILGYTWAVYP
FSWSGLSLDYRDKYGWTALHWAAHYGREKMVATLLSASAKPNLVTDPTSENPGGSTAADL
ASKNGFEGLGAYLAEKALVAHFKDMTLAGNVSGSLQTTTEHINSGNFTEEELYLKDTLAA
YRTAADAAARIQAAFREHSFKVQTKAVESSNPEIEARNIVAAMKIQHAFRNYESRKKLAA

AARIQYRFRSWKMRKDFLNMRRHAIKIQAVFRGFQVRKQYRKIVWSVGVLEKAVLRWRLK
RKGFRGLQVQSSQAVDIKPDGDVEEDFFRASRKQAEERVERSVVRVQAMFRSKRAQEEYR
RMKLEHDNATLEYERASVLNPDIQIG
>NtCAMTA16
MADTRRYLSNQPLDLEQVLQETQHRWLRPAEICEILRNHHKFYLTPEPPVRPPGGSLFLF
DRKVLRYFRKDGHQWRKKKDGKTVKEAHEKLKAGSVDVLHCYYAHGENNENFQRRSYWML
EEKLEHIVLVHYREVIESYRVGASRLQPIHPGQLLENPSSSPCFVSGLIVQESHTSSPSS
VDWKEQALSSELYTGDSKGNEVNPLLVPASGHFLPITSSFSTEKPTGLVEFSRDNFQLNP
QFGSFVSIDAQSSDRNLNVTLQKKFYSGYLNVADLLSSKLTYARLDGGRAVKDVANSRNR
LTITSGEVLEENIHLAPAQIQNISSSQTVVTPDAAVQNSSLEGRLNSDEAGSLKKLDSFG
RWMDREIAVDGNESLLASDSGNYWNTLDNGDKEVARLSCHMQLDTNSLGPFLSQEQLFSI
SDFAPDWAYSGVETKVLIIGTFLGHGKHPTSQKWSCMFGEVEVSAELLTQSIIRCEVPSH
SPGRVPFYVTCSNRLACSEVREFEYREKSSELALALRPSDEVRLQVRLAKLLYSGLNKKF
LDCSSTDCERGKLKTLLCSLKCNIGNASESLEDLLAIIEGNHINFRDTLIQSFMKDKFYE
WLVSRAHEEDKGPNILDDEGQGVIHLVASLGYEWGLVLLTAAGINPNLRDARGRTALHWA
AHYGREDMVIALVKLGVAVCAVDDPTAAFPGGQTAADLASSGGHKGVAGYLAESELTAHL
QSLAINNNALDSICAGLEAEKAFESAAQEVVPLNGTIHDDISLKGSLASVRKSAHAAALI
QAAFRARSFHQRQLRESRNDVSEASVDLVALGSLNKVQKVNHFEDYLHPAAIKIQQKYRG
WKGRREFLKIRNRIVKIQAHVRGHQVRKQYKKFVWSVSIVEKAILRWRRKKPGLRGFQPE
KTSQKELPEFEKNDEYEYLSIGRKQKFAGVQKALARVQSMVRHPEARDQYMRLVAKFDSF
KLDDGGSSI
>NtCAMTA17
MAESGYNINDLVREGHFRWLRPAEVVFILQNHEDQQLANQPPQKPASGSMFLFNKRVLRY
FRKDGHSWRKKKDGRTVGEAHERLKVGNAEALNCYYAHGEKNPNFQRRSYWMLDPAYEHI
VLVHYRDITEGMQIAAFMSQSSPISSTFSLSPSLYSTQHPGFTVFGSESYQQYPNESSPG
SGEVCSDAGINGKGMNISDITGRTEGVSSSPRVEISQALRKLEEQLSLNDDSLEQIDPLY
SEIENSDDVENFVHDNNSLVQIQHKSNNLLLQPHSGESSESQHQLLNLDGNIWKEMLDHC
RSFPAAESPAKCFEKLDENGTLQTSSGVGPIEATESDRWLKFGGKALKSSLTNFKQVEDF
KYPACARINTYGSYSDQYTTIFDQDQIGTSFEDDMSLTIAQKQKFTIHDISPDWGYSSEA
TKIVIVGSFLCNPSEYTWTCMFDDIEVPVQIINEGAIRCQAPPHLPCKVTLCVTTGNRVS
CSEVWEFEYRVKFDDHGQKNLAEVGGACKSSEELLLLVRFVQMLLSDSSVQKGDGSGSSN
DILENSKASEDSWSQVIESLLFGTSTSMVTVDWLLQELLKDRLKQWLSSKLQVKNNQMGY
SFSRKEQGIIHMVAGLGFEWALHPILDAGVGVNFRDINGWTALHWAARFGREKMVASLVA
SSAFAGAVTDPSSQDPFGRTAASIASSCGHKGVAGYLSEVALTSHLSSLTLEENELSKGT
ADVEAERTISSISTTSAATHEDQLSLKDTLAAVRNAAQAAARIQSAFRAHSFRKRRQREA
ARAATTSGDEYCVLSNDVLGLSAASKLAFRNMRDYNSAALAIQKKYRGWKCRKDFLAFRQ
KVVKIQAHVRGYQVRKEYKVCWAVGILEKVVLRWRRRGVGLRGFRLEEEPIEESEDEDIL
KLFRKQKVDAAINEAVSRVLSMVDSPEARQQYHRILEKYRQAKAELGVNSDTVSTAHGDI
SNSDI
>NtCAMTA18
MAESGYNINDLVREGHFRWLRPAEVVFILQNHEDQQLANQPPQKPASGSMFLFNKRVLRY

FRKDGHSWRKKKDGRTVGEAHERLKVGNAEALNCYYAHGEKNPNFQRRSYWMLDPAYEHI
VLVHYRDITEIAAFMSQSSPISSTFSLSPSLYSTQHPGFTVFGSESYQQYPNESSPGSGE
VCSDAGINGKGMNISDITGRTEGVSSSPRVEISQALRKLEEQLSLNDDSLEQIDPLYSEI
ENSDDVENFVHDNNSLVQIQHKSNNLLLQPHSGESSESQHQLLNLDGNIWKEMLDHCRSF
PAAESPAKCFEKLDENGTLQTSSGVGPIEATESDRWLKFGGKALKSSLTNFKQVEDFKYP
ACARINTYGSYSDQYTTIFDQDQIGTSFEDDMSLTIAQKQKFTIHDISPDWGYSSEATKI
VIVGSFLCNPSEYTWTCMFDDIEVPVQIINEGAIRCQAPPHLPCKVTLCVTTGNRVSCSE
VWEFEYRVKFDDHGQKNLAEVGGACKSSEELLLLVRFVQMLLSDSSVQKGDGSGSSNDIL
ENSKASEDSWSQVIESLLFGTSTSMVTVDWLLQELLKDRLKQWLSSKLQVKNNQMGYSFS
RKEQGIIHMVAGLGFEWALHPILDAGVGVNFRDINGWTALHWAARFGREKMVASLVASSA
FAGAVTDPSSQDPFGRTAASIASSCGHKGVAGYLSEVALTSHLSSLTLEENELSKGTADV
EAERTISSISTTSAATHEDQLSLKDTLAAVRNAAQAAARIQSAFRAHSFRKRRQREAARA
ATTSGDEYCVLSNDVLGLSAASKLAFRNMRDYNSAALAIQKKYRGWKCRKDFLAFRQKVV
KIQAHVRGYQVRKEYKVCWAVGILEKVVLRWRRRGVGLRGFRLEEEPIEESEDEDILKLF
RKQKVDAAINEAVSRVLSMVDSPEARQQYHRILEKYRQAKAELGVNSDTVSTAHGDISNS
DI
>NtCAMTA19
MAESGYNINDLVREGHFRWLRPAEVVFILQNHEDQQLANQPPQKPASGSMFLFNKRVLRY
FRKDGHSWRKKKDGRTVGEAHERLKVGNAEALNCYYAHGEKNPNFQRRSYWMLDPESYQQ
YPNESSPGSGEVCSDAGINGKGMNISDITGRTEGVSSSPRVEISQALRKLEEQLSLNDDS
LEQIDPLYSEIENSDDVENFVHDNNSLVQIQHKSNNLLLQPHSGESSESQHQLLNLDGNI
WKEMLDHCRSFPAAESPAKCFEKLDENGTLQTSSGVGPIEATESDRWLKFGGKALKSSLT
NFKQVEDFKYPACARINTYGSYSDQYTTIFDQDQIGTSFEDDMSLTIAQKQKFTIHDISP
DWGYSSEATKIVIVGSFLCNPSEYTWTCMFDDIEVPVQIINEGAIRCQAPPHLPCKVTLC
VTTGNRVSCSEVWEFEYRVKFDDHGQKNLAEVGGACKSSEELLLLVRFVQMLLSDSSVQK
GDGSGSSNDILENSKASEDSWSQVIESLLFGTSTSMVTVDWLLQELLKDRLKQWLSSKLQ
VKNNQMGYSFSRKEQGIIHMVAGLGFEWALHPILDAGVGVNFRDINGWTALHWAARFGRE
KMVASLVASSAFAGAVTDPSSQDPFGRTAASIASSCGHKGVAGYLSEVALTSHLSSLTLE
ENELSKGTADVEAERTISSISTTSAATHEDQLSLKDTLAAVRNAAQAAARIQSAFRAHSF
RKRRQREAARAATTSGDEYCVLSNDVLGLSAASKLAFRNMRDYNSAALAIQKKYRGWKCR
KDFLAFRQKVVKIQAHVRGYQVRKEYKVCWAVGILEKVVLRWRRRGVGLRGFRLEEEPIE
ESEDEDILKLFRKQKVDAAINEAVSRVLSMVDSPEARQQYHRILEKYRQAKAELGVNSDT
VSTAHGDISNSDI
>GmCAMTA1
MAEARHYVPPSQLDIKQIILEAQHRWLRPAEICAILSNHKKFLIASEPAHMPPSGSLFLF
DRKVLRYFRKDGHNWRKKKDGKTVREAHERLKAGSVDVLHCYYAHGEENENFRRRTYWLL
EEELSHIVLVHYRHVKGTKANFTCAKENEETLPYAQQTDKIMPKTEMETSLSSTLHPHSY
QVPSQTMDRSMNSSQASEYEEAESAFNNHASSEFYSFLELERPVEKITPQPADSYSPRPL
TRKSVPNMNCIIESGTDDQEKSPVIPGVNYISLTQDNKIKDIHNFGLTYESPKPLGFSSW
EGILKNNAGSQHVPFQPLFPGTQPDNMGINSKFSQGHEIMVPYLTTSIAKQHENGSLIQA
EGNWQAYDVDSLRMSSWPIDSAYSGSSCDITCSNREQEVNDVDLQKSLEQCLLHPYKQNK

VFMQNDPQEKLLNEKEKIKSDLEANRILDGIEDTYFTFKRTLLDGSPAEEGLKKLDSFNQ
WMSKELADVEESNKPSTSGGYWDTVESENEVGNTTIPSQGHLDTYVLDPSVSHDQLFSII
DYSPSWAFEGSEIKVIISGRFLRSQHEAEQGKWSCMFGEVEVPAEIIAKGVLCCHTPPHK
AGRVPFYVTCSNRLACSEVREFDFQVNYTPEVNTTGENRGSTFDTFSIRFGELLSLGHAF
PQNSDSISVSEKSQLRSKINSLLREEEDDWDKLLKLTQEEDFSPENLQEQLLQNLLKDKL
HAWLLQKITEEGKGPNILDEGGQGVLHFASALGYDWALEPTIVAGVNVNFRDVNGWTALH
WAAFCGRERTVAFLISLGAAPGALTDPCPEHPSGRTPADLASANGHKGIAGYLAESSLSA
HLTTLDLNRDAGENSGAKVVQRVQNIAQVNDLDGLSYELSLKDSLAAVRNATHAAARIHQ
VFRMQSFQRKQLKEYDDDKLGLSDERALSLVKMNMKSHKSGPRDEPVHAAAVRIQNKFRS
WKGRREFLMIRQRIVKIQAHVRGHQVRKSCGKIIWSVGILEKVILRWRRKGSGLRGFKPE
ANSEGTMIQDVSSTDDDYDVLKEGRKQTEQRLQKALARVKSMVQYPEARDQYHRLLNVVT
EIQENQVKHESSYNNSEEPREFGDLNDLEALLDEDIFMPTAT
>GmCAMTA2
MAEARLYAPPSQLDIKQIILEAQHRWLRPAEICAILGNYKKFRIAPEPAHMPPSGSLFLF
DRKVLRHFRKDGHNWRKKKDGKTVREAHERLKAGSVDVLHCYYAHGEENENFQRRTYWLL
EEELSHIVLVHYRQVKGTKANFTSAKENEESLPYAQQTDKIMPQTEMDTSLSSTLHPHSY
QVPSKTVDTSMNSAQTSEYEEAESAFNNHASSEFYSFLELQRPVEKISPQPADFYSPRPL
INDQEKLPIIPGVNYISLTQDNKNKDILNAGLTYESPKPLGFSSWEGILENNAGSQHVHF
QPLFPGTQPDNMGINSNFSQGEEIMVPYLTTSIAKQHENGSIIKAEGNWQVYDVDSLRMS
SWPIDSAYSGSTCEVSCSNCEQEVNDVDFQKSLEQCLLHSHKQNKVLMQNDLQEKLLNEK
EKIKSNLEAYGIEDTYLSFKRTLLDGPPAEEGLKKLDSFNQWMSKELGDVEESNKPSTSG
GYWDTVETENEVGNTTIPSQGHLDTYVLDPSVSHDQLFSIIDYSPSWAFEGSEIKVIISG
EFLRSQHEAEQCKWSCMFGEVEVPAVIIAKGVLCCHTPPHKAGRVPFYVTCSNRLACSEV
REFDFQVHYTPEDTTGENRGSTFDTFSIRFGELLSLGHAFPQNSDSISVSEKSQLRSKIN
SLLREDDDDWDKLLKLTQEKDFSPENLREQLLQNLLKDKLHAWLLQKITEEGKGPNVLDE
GGQGVLHFAAALGYDWALEPTIVAGVNVNFRDVNGWTSLHWAAFCGRERTVAFLISLGAA
PGALTDPCPEHPSGRTPADLASANGHKGIAGYLAESSLSAHLTTLDLNRDAGENSGAKVV
QRLQNIAQVNDLDGLSYELSLKDSLAAVCNATQAAARIHQVFRMQSFQRKQLKEYDDDKL
GLSDERALSLIKMNVKSHKSGPRDEPVHAAAIRIQNKFRSWKGRREFLMIRQRIVKIQAH
VRGHQVRKSCGKIIWSVGILEKVILRWRRKGSGLRGFKPEANSEGTMIQDVSSTDDDYDV
LKEGRKQTEQRLQKALARVKSMVQYPEARDQYHRLLNVVTEIQENQVKHESSSNNSEEPR
EFGDLNDLEALLDEDIFMPTAT
> GmCAMTA3
MAEGASYGLRRPLDIQQLQFEAQHRWLRPAEICEILRNYRMFHITSEPHNRPPSGSLFLF
DRKVLRYFRKDGHNWRKKKDGKTVKEAHEKLKVGSVDVLHCYYAHGEENENFQRRSYWML
EPDMMHIVFVHYLEVKGNKNIVVNNEGDEVPTDSQKVTSPSSSLPTHHSCVSSLSTDSVS
PTTSLMSLHEDADSEDIHHASSGLHPLHESQHSGNSPLTEKIGAGSNSSYLMHPFSGDNE
QSSISGTDYVPVVHGDKFRGNDTAYTDGQKPHGMAPWGTVLQSTAKLHNDPSLASFPSIL
PSSMGDVLEQEHTIFGDLLMSKSGLTEEAESSQSLQSNWQIPFEDNSGGMPMLTQTQSFG
LQFRSDYGTGLLGNETRNASSEIAPILYSFHGEPKEQPMQQNYPQELEDGQSQHALKSNS
ANKVPDEETINYGLTVKSTLLDRDESLKKVDSFSRWITKELGEVADLNMQSSPGISWSTD

ECQHVIDDTSLSPSLSQDQLFSINDFSPKWAYAESEIEVLIIGSFLKSQPEVTTCNWSCM
FGEVEVPAEVLADGILCCQAPCHKVGRVPFYVTCSNRLACSEVREFDFREGFARNVDFAD
FYISSTEMLRHLRLEDFLSLKPVDPSNHSFEGDMEKRNLIFKLISLREEEDYSIKDEVTR
ELDISQHMVKEHLFHRQFKEKLYSWLLHKVTENGKGPNVLDEDGQGVLHLAAFLGYDWAI
NPIISAGVNINFRDVNGWTALHWAASCGRERTVAVLVSMGADCGALTDPSPAFPSGRTAA
DLASSYGHKGISGFLAESSLTHHLETLTMDDQKGGQQEISGMKVVQTVSERSATPVHYCD
IPDAICLKDSLTAVRNATQAADRIHQVYRMQSFQRKQLTQYEGDDELGLSDQQALSLLAS
RACKSGQGDGLANAAAVQIQKKFRGWKKRKEFLMIRQRVVKIQAHVRGHQIRKQYKPIIW
SVGILEKVILRWRRKGSGLRGFRPNAINKVPNQQNDSLKEDDYDYLKEGRKQKEEKIQKA
LSRVKSMVQYPEARAQYRRLLNVVEDFRQTKASNKGLINSEETVDGVEDLIDIDMLLDDD
NFIPIAFD

> GmCAMTA4

MSERSSFGLGPRLDLQQLQLEAQHRWLRPAEICEILRNYRMFQITSEPPNRPPSGSLFLF
DRKVLRYFRKDGHNWRKKKDGKTVKEAHEKLKVGSVDVLHCYYAHGEENENFQRRSYWML
ELDMMHIVFVHYLDVKVNKTNIGGKTYSDEVTSDSQKSSSLSSGFPRNYGSMPSGSTDSM
SPTSTLTSLCEDADSEDIHQASSGLHSYRESQNLGNDRPMDKIHARSNSSYLMHPFSDNH
GQLPVSGAEYIPHVQGNKSRASDTTYIEGQRAHGIASWDNAMEQSAGKHADPSLVSSTSI
PSSAMGNILDKNHTVPGNLLGHKIALTEVERGAQPVQSNWQIPFEDNTGELPNWGFTQSL
GLEFGSDYGTSLLGDVTNNAGPEIDPELFTFNGELKEQYTHGQSQPALKSNSAYEVPGEA
SINYALTMRRGLLDGEESLKKVDSFSRWMTKELAGVDDLHMQSSPGISWSTDECGDVIDD
TSLHLSLSQDQLFSINDFSPKWAYAESEIEVLIVGTFLKSQPVVAKCNWSCMFGEVEVPA
EVLADGILCCQAPPHKIGRVPFYVTCSNRFACSEVREFEYREGFDRNINFPDFFNNSSEM
ELHLRLVGLLSLNSMHTLNQVFEGDMDKRNLIFKLISLKEEEEYSSKEETTAEMDISQQK
LKEHMFHKQVKEKLYSWLLHKVTETGKGPLVLDEEGQGVLHLIAALGYDWAINPIITAGV
NINFRDVNGWTALHWAAFCGRERTVAVLVSMDAAAGALTDPCPEFPLGRTPADLASSKGH
KGISGFLAESLLTSHLESLTMDENKDGRKETSGMKVVQTVSERTATPVLNGDIPDDICLK
DSLNAVRNATQAADRIYQVFRMQSFQRKQLALYEDDEFGLSDQQALSLLASKACRSGQGE
GLANAAAIQIQKKFRGWTKRKEFLIIRQRIVKIQAHVRGHQVRKQYKPIIWSVGILEKVI
LRWRRKGSGLRGFRPASQNKVPEQPSESPKEDDYDYLKEGRKQSEVKFKKALSRVKSMVQ
YPEARAQYRRVLNVVEDFRQTKGGNLNLINSEETVDGVEDLIDIDMLLDDENFLPIAFD

> GmCAMTA5

MAERSCFGLGPRLDLQQLQLEAQHRWLRPAEICEILRNYQMFQITSEPPNGPPSGSLFLF
DRKVLRYFRKDGHNWRKKKDGKTVKEAHEKLKVGSVDVLHCYYAHGEENENFQRRSYWML
EPDMMHIVFVHYLDVKVNKTNVGGKTYSDEVTSDSQKSSSLSSGFPRNYGSVPSGSTDSM
SPTSTLTSLCEDADSEDIHQASSGLHSYRESQNLGNDRPMDKIDARSNSSYPMHPFSGDH
GQLPVSGAEYIPHVLGDKSRASDTTYIEGQRAQGIASWDNTMEQSAGEYADPSLVSSTTI
PSSAVGNILEENHTVPGKLLGRKNALTEEERGSQPVQSNWQIPFEDNTGELPNWGFTQSL
GLEFGSDYGASLLGDVTNNAGPEIVPELFTFNGELKEQSVHQNFSKLYTHGQSQPTLKSN
SEYEVPGEASINYALTMRRGLLDGEESLKKVDSFSRWMTKEFAGVDDLHMQSSPGISWST
DECGDVIDDTSLNLSLSQDQLFSINDFSPKWAYAESEIEVLIVGTFLKSQPVVAKCNWSC
MFGEVEVPAEVLADGILCCQAPPHKIGRVPFYVTCSNRFACSEVREFEYREGFDRNIQFA

DCFNNSTEMVLHLRLVGLLSLNSVRTSNQVFEGDMDKRSLIFKLISLKEEEEYSSKEETT
AEMDISKHKLKELMFHKQVKEKLYSWLLHKVTETGKGPLVLDEEGQGVLHLIAALGYDWA
INPIITAGVNINFRDVNGWTALHWAAFCGRERTVAVLVSMGAAAGAWTDPCPEFPSGRSP
ADLASSKGHKGISGFLAESLLTGHLESLTMDENKDGRKETSGTKVVQTASERTATPVLYG
DIPDAICLKDSLNAVRNATQAADRIYQVFRMQSFQRKQFAQYEDDEFGLSDQQALSLLAS
KTCKSGQGEGLANAAAIQIQKKFRGWTKRKEFLIIRQRIVKIQAHVRGHQVRKQYKPIIW
SVGILEKVILRWRRKGSGLRGFRPAALNKVPEQPSESPKEDDYDYLKEGRKQSEVKFKKA
LSRVKSMVQYPEARAQYRRVLNVVEDFRQTKGGNLNLINSEETVDGVEDLIDIDMLLDDE
NFLPIAFD
>GmCAMTA6
MAEGASYGLRRSLDIQQLQFEAQHRWLRPAEICEILRNYRMFHITSEPHNRPPSGSLFLF
DRKVLRYFRKDGHNWRKKKDGKTVKEAHEKLKIGSVDVLHCYYAHGEENENFQRRSYWML
EPDMMHIVFVHYLEVKGNKNIVVNTEGDEIPSDSQKVTSSSSSLPTHHSSVPSLSTDSVS
PTTSLMSLREDADSEDIHQASSGLRPLYESQHSGNGPLTEKIGAGSNSSYLIHPFSGDYE
QSSISGTDYIPVVHGDKFRGNDTAYIDGQKTHDVATWSTVLQSTAKLHNDPSLASSPSIP
SSSMGDVLEQEHTIFSDLLMSKSGLTEVAESSQSLQSNWQIPFEDNSGGMPMLTQTQTFG
LQFRSDYGTGLLGNETRNACSESAAILYSFNGEPKEQPMQQNYLQELEDGQSQHALKSNS
ANKVPDEETINYGLTVKRTLLDKDESLKKVDSFSRWITKELGEVADLNMQSSPGISWSTD
ECQHVIDDTSLSPSLSQDQLFSINDFSPKWAYAESEIEVLIIGSFLKSQPEVTTCNWSCM
FGEVEIPAKVLADGILCCQAPRHKVGRVPFYVTCSNRLACSEVREFDFREGFARNVDFAD
FYNSSEILLHLRLEDFLSLKPVDPSNHSFEGDMEKRNLIFQLISLREVEEYSIKDEVTTE
LDISQHMVKEHLFHKQFKEKLYSWLLHKVTESGKGPNVLDEDGQGVLHLAAFLGYDWAIN
PIISAGVNINFRDVNGWTALHWAASCGRERTVAVLVSMGADCGALTDPSPSSPAGRTAAD
LASSYGHKGISGFLAESSLTHHLETLTMDDQKGGRQEISGMKAVQTVSERSATPVHFGDM
PDLCLKDSLTAVRNATQAADRIHQVYRMQSFQRKQLTQYESDELGLSDQQALSLLASRAC
KSGQGDGLANAAAVQIQKKFRGWKKRQEFLMIRQRVAHVRGHQVRKQYKPIIWSVGILEK
IILRWRRKGSGLRGFRPNVINEVPDQQNNSLKEDDYDYLKEGRKQKEEKIQKALSRVKSM
AQYPEARAQYRRLLNVVEDFRQTKASNEGLINSEETVDGMEDLIDIDMLLDDDNFIPIAF
D
>GmCAMTA7
MAETTKYIPNSQLELEEILNEAEHRWLRPAEICEILRNHKKFKLTPDPPVMPPAGSLFLF
DRKALRYFRKDGHRWRKKKDGKTVREAHEKLKAGSVDVLHCYYAHGEDNEYFQRRSYWML
DEQLEHIVLVHYREIKEGCKSGISHLPVVPVTLVGSSQNTSVLSSTKINSPISLVQTSFT
SSANKVYQNGRASEHEDVNSKNGPQASSHAQPISNYVLHSAPWLTHEAAGFSELLRNPLI
SSWPSSFPSYSPGTGLSPWTSIQNSSRNTINMHDGKHHVEASEADLTVRKLSNAGLDSVH
RMQDGVIFRDRLITDMCVQPVIDLPTVNQVKNEHGLDSFHAQVHDHNDHPVVATTKILVE
QKLQDGGLYNDESEQVEYGEMKKLDSFGRWMDKEIGGDCDNSLMASDSGNYWSTLDAHSE
DKEVSSLRHMQLDVDSLGPSLSQEQLFSIHDFSPDWAYTGVRTKVLIVGTFLGSKKPSSE
TKWGCMFGEIEVSAEVLADNVIRCQTPLHSPGRVPFYITCSNRLACSEVREFEFDENPTK
FLGPEGIKISPEEEVRLQMRLLKLVDLGPDNKWLKCSVSECEKCKLKGTMYSVRDDSGVF
EETFQIDGIGHINHRDILFQRLVRDKLYEWLIYKVHEGGKGPHVLDDEGQGVIHLAAALG

YVWAMAPLVAAGISPNFRDSRGRTGLHWASYFGREETVIVLVQLGATPGAVEDPTSAFPR
GQTAADLGSSRGHKGIAGYLAEADLTNQLSVLTVKENETGNIATTIAANSALQSVEDDSS
SMTMDEQHYLKESLAVFQKSAHAAASILAAFRARSFCQRQLAQSSSDISEVLDVVADSLS
KVQNKGHFEDYLHFAALKIQKRYRGWKGRKDFLKIRDRIVKIQAHIRGHQVRKQYKKVVW
SVSIVEKAILRWRRKGAGLRGFRVGQPVGVVVKDAEKSDEYEFLSIGRRQKSDDVKKALD
RVKSMVRNPEARDQYMRLIMKYEKFKIDDGGSSQSQHVG

>GmCAMTA8

MMSHNLTGQLVSAEIHGFHTLEDLDVSNTMEEAKSRWLRPNEIHAILCNHKYFKINVKPV
NLPKSGTIVLFDRKMLRNFRKDGHNWKKKTDGKTVKEAHEHLKVGNEERIHVYYAHGQDN
PTFVRRCYWLLDKNLEHIVLVHYRDTQELQLQGSPATPVNSNSSSASDPAASWIPSEDLD
SGVNSAYAVELNDNLTAKSHEQRLHEINTLEWDDLVVPNVNTSTTSNGGNVPYSFQENQS
LLSGRFGNVSSNPSAEIPSFGNLTQPVSGSNSAPYSFPDSAILLKNSPISSGGVDTLGTL
VNEGLQSQDSFGTWMNIISDTPCSIDESALKASISSVHVPYSSLVADNLQSSLPEQVFNL
TEVSPTWASSTEKTKVLVTGYFHNNYENLAKSNLLCVCGDVSVPVEIVQVGVYRCCVPPH
SPGLVNLYLSFDGHKPISQVVNFEYRTPILHEPTASMEEKYNWNEFRLQMRLAHLLFASD
TSLNIFSSKVSPNALKEARRFSFKTSYISKSWQYLMKSIDDNTIPFSKVKDSLFETALKN
KLKEWLLERIILGRKSTEYDAQGQGVIHLCAMLGYSWAISLFSWSGLSLDFRDKFGWTAL
HWAASYGMEKMVATLLSCGARPNLVTDPTPQYPGGCTAADLAYMKGCDGLAAFLSEKSLV
EQFNEMSLAGNISGSLETSSTDPVNAENLTEDQLYVKETLAAYRISAEAAARIQAAFREH
SFKLRYKAVEIISPEEEARQIVAAMRIQHAFRNYESKKKMTAAARIQHRFRTWKYRREFL
NMRHQAIKIQAAFRGFQARKQYRKIIWSVGVLEKVILRWRLKRKGFRGLQVNPAREETQE
SDSIAEEDFFRTGRKQAEERIERSVIRVQAMFRSKKAQEEYRRMKLTHNQAKLELELEEF
LDSEVDMLPKT

>GmCAMTA9

MMMSHNLTGQLVGAEIHGFHTLDDLDVSNTMEEAKSRWLRPNEIHAILCNHKYFKINAKP
VNLPKSGTIVLFDRKMLRNFRKDGHNWKKKTDGKTVKEAHEHLKVGNEERIHVYYAHGQD
KPTFVRRCYWLLDKSLEHIVLVHYRDTQELQLQGSPATPVNSNSSSVSDSAASWIPSDDL
DSGVNSAYAVELNDSLTAKSHEQRLHEINTLEWDDLVVSNANTSTTSNGGNVPYSFQQNQ
SLLNGSFGNVSSDPSAEIPSFGNLTQLVSGSDSAPYSFPESADLLKSSPLSSGGVDTLGT
LVNEGLQSQDSFGTWMNIMSDTPCSIDESALEATTSSVHVPYSSLVADNKQSSLPEQVFN
LTEVSPVWASSTEKTKVLVTGYFHNNYQHLAKSNLLCVCGDVSVPVEIVQVGVYRCWVSP
HSPGLVTLYLSFDGHKPISQVVNFEYRTPILHEPTALIEEKYNWDEFRLQMRLAHLLFAS
DKSLNIFSSKVSTNALKEARRFSFKTSYISKSWQHLMKSIDDKTIPFSQVKDALFETSLK
NKLKEWLLERIILGSKSTEYDAQGQAAIHLCAMLGYNWAISLFTWSGLSLDFRDKFGWTA
LHWAAYYGIEKMVATLLSCGARPNLVTDPTPQYPGGCTAADLAYVKGCDGLAAYLSEKSL
VEQFNDMSLAGNISGSLETSSTDPVNAANLTEDQLYLKETLEAYRTAAEAAARIQAAFRE
HSFKLRYQAVEIMSPEEEARQIVAAMRIQHAFRNYESKKKMAAAARIQLRFRTWKYRREF
LNMRRQAIKIQAAFRGFQARKQYRKIVWSVGVLEKVILRWLLKRKGFRGLQVNPAEEETQ
ESDTIAEEDFFRTSRKQAEERVERSVIRVQAMFRSKKAQEEYRRMKLTHNQAMLDELEEF
LNSEDDMLPKT

>GmCAMTA10

MTPGYEYDINDLHQEAQARWLKPAEVMYILQNHEKFQFTQEPPQQPTSGSLFLFNKRVLR
FFRKDGHNWRKKRDGRTVGEAHERLKVGNVEALNCYYAHGEQNPTFQRRSYWMLDPAYDH
IVLVHYRNTSEGKLSSGAGAQLSPSSSSVYTQSPSPYSTQNPGSTSILGDSYEPNQSFSS
PGSTEVTSDMFVLNNKMGHMDGTDTESGTSPELEVTQALRRLEVQLSLNEDNFEDIVSFG
SKHETTHDSNPQHDQRVISNQEQSAAFSGPDDQGLFYDGYNGRQGDGGEFYHELIDHGYP
DGNEKALWTEVLESCKSSSAVKLPQKNVYMPVENLENSVSSARRVPVSNQENSHWLNFNS
NNSENSVFSQPQGVDEVKFPVYSSMVETQVINSDYYETLFDQSQIGAPPDANSSLTVAQK
QKFTIKTISPEWGYATETTKVIVVGSLLCHPSDSAWACMFGDVEVPVEIIQDGVISCEAP
SHLPGKVTLCITSGNRESCSEVREFEYRDKTNSCTQCTQSETEATRSPEELLLLVRLEQM
LLSASTIKNDNIESGIPLIKQKADDDSWSHIIEALLVGSGTSTGTVDWLLEELLKDKLQQ
WLSCRSQEKDEETGCSLSKKEQGIIHMVAGLGFEWALNPILTCGVNINFRDINGWTALHW
AARFGREKMVASLIASGASAGAVTDPNAQDPTGKTAASIAAGNGHKGLAGYLSEIAVTSH
LSSLTLEESELSKSSAELQADMTVNSVSKENLTASEDQASLKDTLAAIRNVTQAAARIQS
AFRSHSFRKRRAREVAASAGGIGTISEISAMSKLAFRNSREYNSAASAALSIQKKYRGWK
GRKDFLALRKKVVKIQAHVRGYQVRKHYKVIWAVGILDKVVLRWRRKGAGLRGFRQEMDI
NENENEDEDILKVFRKQKVDVEIEEAVSRVLSMVDSPDAREQYHRMLEKYRQAKAELAGT
SDEASLSTSVGDDLFIDDFYPFP
>GmCAMTA11
MPPGLEYNIDDLFQEAKRRWLKPVEVLYILRNHDQCEFTHQPPHQPAGGSLLLFNRRIMR
FFRKDGHNWRKKKDGKTVGEAHERLKVGNVEILNCYYAHGEENRTFQRRSYWMLEPEYDH
IVLVHYRETSEGKSKSEHVTQLSSGSSPVFSQSHSSYTTHNPGTASMFGDSCEPNQKFSS
SGSLEDTSEAQALRQLEEQLSLNEDIFNEIALDLIPGQDQRVVYKQDNSVALSGPNDPGQ
PCDGYNGREDDSGTYYHDFLDDCPGGNEKTIYWTEVLESCKPLSVTKLPDQHAYDAIENG
KSLFSSGRGMIANREKNQWLNSNSNNVENSVFLFPQDIGVKFPPYSMVETPGTNYDYYET
CFDQFQNQEPLGVDSSFTVVQKQKFTIRAVSPEYCYATETTKVIIIGSFLCHDSDSTWAC
MFGDVEVPAEIIQDGVICCEAPSYLLGKVNLCVTSGNRVPCSEVRGFEFRNKTTSCTRCN
SLETEGSKSLEDLLLLVRFAEMLLSASTTKDDRIESGSYLSTEQKDDDDSWSHIIIDTLL
DGTRTSSDTVNWLLEELLKDKLQLWLSNRRDEGTGCSFSRKEQGIIHMISGLGFEWALSP
ILSCGVNINFRDINGWTALHWAARFGREKMVASLIASGASAGAVTDPSSQDPTGKTAASI
AASHGHKGLAGYLSEVDLTSHLSSLTLEESELSKGSSELEAELTVSSVSKENLVASEDQV
SLQAFLDAVRNAAQAAARIQAAFRAHSFRKRKEREAAADAGLDGYCIDAGSIDNNISVLS
AVSKLSSQSCRDYNLAALSIQKKYRGWKGRKEFLALRQKVVKIQACVRGYQVRKQYKLIL
WAVGILDKVVLRWRRKRIGIRSVRQEMESNEEESDDEDFLSVFRKEKVNAAIEKALKQVL
SMVHSSGARQQYRRLLLLYRQAKAKTERGSTSDEAPLSTSEEEVSNMEDDDLCQFWETFW
PS
>GmCAMTA12
MANNLAAQQLVGSEIHGFHTLQDLDVGSIMEEARTRWLRPNEIHAMLCNYKYFTINVKPV
NLPKSGTIVLFDRKMLRNFRKDGHNWKKKKDGKTVKEAHEHLKVGNEERIHVYYAHGQDN
PNFVRRCYWLLDKSMEHIVLVHYREIQEMQGSPVTPVNSHSSSVSDPPAPWILSEEIDSG
TTTAYAGDTSANINVKSHELRLHEINTLDWDDLVDANDHNTTTVPNGGTVPYFDLQDQIL
LNDSFSNVANNLSADIPSFGSLTQPIAGSNSVPYNFSSVNLQTMDDQANPHEQRNNTVSL

SGVDSLDTLVNDRLQSQNSFGMWVNPIMSDSPCSVDDPALESPVSSVHEPYSSLIVDSQQ
SSLPGQVFTITDVSPTCVSSTEKSKVLVTGFFHKDYMHLSKSNLLCVCGDVSVPAEIVQV
GVYRCWVSPHSPGFVNLYMSIDGHKPISQVVNFEYRTPALHDPAVSMEESDNWDEFQLQM
RLAYLLFKQLNLDVISTKVSPNRLKEARQFALKTSFISNSWQYLIKSTEDNQIPFSQAKD
ALFGIALKSRLKEWLLERIVLGCKTTEYDAHGQSVIHLCAILGYTWAVSLFSWSGLSLDF
RDRSGWTALHWAAYCGREKMVATLLSAGAKPNLVTDPTPQNPGGCTAADLAYMRGHDGLA
AYLSEKSLVQHFNDMSLAGNISGSLETSTTDPVISANLTEDQQNLKDTLAAYRTAAEAAS
RIHAAFREHSLKLRTKAVASSHPEAQARKIVAAMKIQHAFRNHKTKKVMAAAARIQCTYR
TWKIRKEFLNMRCQAVKIQAAFRCFQVRKHYCKILWSVGVVEKAVLRWRLKRRGFRGLQV
KTVEAGTGDQDQQSDVEEEFFRAGRKQAEERVERSVVRVQAMFRSKKAQEEYRRMKLALD
QAKLEREFERLLSTEVDMQLKT
>GmCAMTA13
MANNLAVQLVGSEMHGFHTLQDLDVGSIMEEARTRWLRPNEIHAMLCNYKYFTINVKPVN
LPKSGTIVLFDRKMLRNFRKDGHNWKKKKDGKTVKEAHEHLKVGNEERIHVYYAHGQDNP
NFVRRCYWLLDKSMEHIVLVHYRETQEMQGSPVTPVNSHSSSVSDPPAPWILSEEIDSGT
TTAYTGDMSNNINVKSHELRLHEINTLEWDDLVDTNDHNASTVPNGGTVPYFDQQDQILL
NDSFGNVANNLSAEIPSFGNLTQPIAGSNRVPYNFSESVTLQTMDNQANPHEQKNNTVSL
SGVDSLDTLVNDRLQSQDSFGMWVNHIMSDSPCSVDDPALESPVSSIHEPYSSLVVDSQE
SSLPEQVFTITDVSPTCVSSTEKSKVLVTGFFLKDYMHLSKSNLLCVCGDVSVPAEIVQV
GVYRCWVSPHSPGFVNLYLSIDGHKPISQVVNFEYRTPALHDPAVSMEESDNWDEFRQQM
RLAYLLFAKQLNLDVISSKVSPNRLKEARQFALKTSFISNSWQYLIKSTEDNQIPFSQAK
DALFGITLKNRLKEWLLERIVLGCKTTEYDAHGQSVIHLCAILGYNWAVSLFSWSGLSLD
FRDRFGWTALHWAAYCGREKMVATLLSAGAKPNLVTDPTPQNPGGCTAADLAYMRGHDGL
AAYLSEKSLVQHFNDMSLAGNISGSLETSTTDPVNPANLTEDQQNLKDTLTAYRTAAEAA
SRIHAAFREHSLKLRTKAVASSNPEAQARKIVAAMKIQHAFRNHETKKMMAAAARIQCTY
RTWKIRKEFLNMRRQAVKIQAAFRCFQVRKHYRKILWSVGVVEKAVLRWRLKRRGFRGLQ
VKTVDAGTGDQDQQSDVEEEFFRTGRKQAEERVERSVVRVQAMFRSKKAQEEYRRMKLAL
NQAKLEREYEQLLSTEVDMQL
>GmCAMTA14
MAGLEYSIDDLFQEAKRRWLKPVEALYILRNHDQCKFTHQPPHQPAGGSLFLFNRRIMRS
FRKDGHNWRKKKDGKTVGEAHERLKVGNVEILNCYYAHGEENRTFQRRSYWMLEPEYDHI
VLVHYRETSEGKSNSEHVTQLPSEVTFEAQALRQLEEQLSLNDDGFNEIALDLVSGQDQR
VVYKQDKSAALSGPNDLGQPCDGYNGRQDDSGTYYHDFLDDCPGGNEKTIYWTKVLESCK
PLSVTKLPDQHAYEAIGNENTLFSSGRGVIANLENNQWLNSNSNNIENYGGVKFPPYSLA
ETPGANSDYYETFFDQFQNQGPLGVDSSLTVVQKQKFTIRAVSPEYCYSTETTKVIIIGS
FLCHDSDSTWACMFGDVEVPAEIIQDGIICCEAPSNHLGKVNLCITSGNRVPCSEMREFE
FRNKTTSCTRCNSLETEGSKSPEDLLLLVRFAEMLLSSSTTKDDRIESGSHLSTEQKDDD
DSWSHIIDTLLDSTRTPSDAVKWLLEELLKDKLQLWLSNRRDEGTGCSLSKKEQGIIHMV
SGLGFEWALNPILSCGVNINFRDINGWTALHWAARFGREKMVASLIASGASAGAVTDPSS
QDPTGKTAASIAASHDHKGLAGYLSEVDLTSHLSSLTLEESELSRESSELEAELTVSSVS
EENLVASEDQVSLKASLDAVRNAAQAAARIQAAFRAHSFRKRKERDAAATVLDGYCIDAG

SIDNNISVLSAMSKLSSQSWRDYKAALSIQKKYRNWKGRIEFLALRQKIVKIQACVRGYQ
VRKQYKLILWAVGILDKVVLRWRRKRIGIQSVRQEMESNEEESDDADFLNVFRKEKVNAA
IEKALKRVLSMVHSTGARQQYRRLLSLYRQAKIEHGSTSDEAPLSTSEENASNMEDDDLC
QFLDTFFWPS
>GmCAMTA15
MTPGYEYDINDLHQEAQARWLKPAEVMYILQNHEKFQFTQEVPQQPTSGSLFLFNKRILR
YFRRDGHNWHKKSGGRTVGEAHERLKVLNVEALNCYYARGEQNPAFQRRSYWMLDPAYEH
IVLVHYRNTSEGKLSSGAGAQLSPSSSVYTQSPSPYSTQNPGSTSILGDSYEPNQSFSSP
GSTKVTSEIFVLNNKMGHMDWADTESGTSSELEVTQALRRLEVQLSLNEDNFEDIVSFGS
KHETVHDSNPKHDQRVISNQEQSAAFSRPDDQGLFYDGCNGRQDHGYPDANEKALWTEQL
ESHKSSSAVKLPQKNVYMPAENENSVSSARRVPVSNQENSHWLNFNCNNSENSVFSQPQG
VDEVKFPAYSSMLETQVINSDYYETLFDQSQIGAPPDANSSLTVAQKQKFTIKTISPEWG
YATETTKVIVVGSFLCHPSDSAWACMFGDVEVPIEIIQDGVISCEAPSHLPGKVTLCITS
GNWESCSEVREFEYHDKTNSCTRCTQSETEATRSPEELLLLVRLGQMLLSASTIKNDNIE
SGIPLIKPKADDDSWSHIIDALLVGSGTSSGTVDWLLEELLKDKFQQWLSFRSREKDEET
GCSLSKKEQGIIHMVAGLGFEWALNPILTCGVNINFRDINGWTALHWAARFGREKMVASL
IASGASAGAVTDPNAQDPTGKTAASIAASSGHKGLAGYLSEIAVTSHLSSLTLEESESSK
SSAYLQADRTVNSVSKENLTANEDQASLKDTLAAIRNVTQAAARIQSAFRSHSFRKRRAR
EATASTGGIGTISEISAMSKLAFRNSHEYNSAALSIQKKYRGWKGRRDFLALRQKVVKIQ
AHVRGYQVRKHYKVIWAVGILDKVVLRWRRKGAGLRGFRQEMDINENEDEDILKVFRKQK
LDVEIEEAVSRVLSMVDSPDAREQYHRMLEKYRQAKAELAGTSDEASLSTSVGDDLFMDD
FYPFP
